# Cold-induced lipid dynamics and transcriptional programs in white adipose tissue

**DOI:** 10.1186/s12915-019-0693-x

**Published:** 2019-09-17

**Authors:** Ziye Xu, Wenjing You, Yanbing Zhou, Wentao Chen, Yizhen Wang, Tizhong Shan

**Affiliations:** 1 College of Animal Sciences, Zhejiang University, 866 Yuhangtang Road, Hangzhou, 310058 China; 2 The Key Laboratory of Molecular Animal Nutrition, Ministry of Education, Zhejiang University, 866 Yuhangtang Road, Hangzhou, 310058 China

**Keywords:** Lipid metabolism, RNA sequencing, Lipidomics, Cold exposure, WAT browning, Fatty acid, Triacylglycerol, Glycerophospholipid, Sphingolipid

## Abstract

**Background:**

In mammals, cold exposure induces browning of white adipose tissue (WAT) and alters WAT gene expression and lipid metabolism to boost adaptive thermogenesis and maintain body temperature. Understanding the lipidomic and transcriptomic profiles of WAT upon cold exposure provides insights into the adaptive changes associated with this process.

**Results:**

Here, we applied mass spectrometry and RNA sequencing (RNA-seq) to provide a comprehensive resource for describing the lipidomic or transcriptome profiles in cold-induced inguinal WAT (iWAT). We showed that short-term (3-day) cold exposure induces browning of iWAT, increases energy expenditure, and results in loss of body weight and fat mass. Lipidomic analysis shows that short-term cold exposure leads to dramatic changes of the overall composition of lipid classes WAT. Notably, cold exposure induces significant changes in the acyl-chain composition of triacylglycerols (TAGs), as well as the levels of glycerophospholipids and sphingolipids in iWAT. RNA-seq and qPCR analysis suggests that short-term cold exposure alters the expression of genes and pathways involved in fatty acid elongation, and the synthesis of TAGs, sphingolipids, and glycerophospholipids. Furthermore, the cold-induced lipid dynamics and gene expression pathways in iWAT are contrary to those previously observed in metabolic syndrome, neurodegenerative disorders, and aging, suggesting beneficial effects of cold-induced WAT browning on health and lifespan.

**Conclusion:**

We described the significant alterations in the composition of glyphospholipids, glycerolipids, and sphingolipids and expression of genes involved in thermogenesis, fatty acid elongation, and fatty acid metabolism during the response of iWAT to short-term cold exposure. We also found that some changes in the levels of specific lipid species happening after cold treatment of iWAT are negatively correlated to metabolic diseases, including obesity and T2D.

**Electronic supplementary material:**

The online version of this article (10.1186/s12915-019-0693-x) contains supplementary material, which is available to authorized users.

## Background

Adipose tissues play crucial roles in lipid and glucose metabolism. Brown adipose tissue (BAT) protects against hypothermia by its unique expression of mitochondrial uncoupling protein 1 (UCP1), which drives adaptive non-shivering thermogenesis at low ambient temperature in mammals [1]. In this process, BAT transforms the chemical energy stored in lipids into heat. In contrast, white adipose tissue (WAT) serves as a site for the energy storage, and uncontrolled expansion causes overweight and obesity. The third type of adipocytes called beige or brite adipocytes have recently been identified from WAT and exhibit the characteristics of BAT in response to various stimuli, including cold, dietary interventions, or pharmacological treatments [2–5]. This adaptive emergence of beige or brite adipocytes from WAT is known as browning and is commonly observed in the subcutaneous deports of WAT, such as in the inguinal WAT (iWAT) [6]. Similar to classical brown adipocytes, beige adipocytes can also burn lipids to produce heat through a classical UCP1-dependent mechanism [7]. Unlike BAT, which arises from the myogenic factor 5 (Myf5)-myogenic lineage [8], beige adipocytes originate from both Myf5^+^ and Myf5^−^ lineages and uniquely express several specific cell surface markers, such as transmembrane protein 26 (Tmem26) and CD137 [4, 9, 10].

BAT and beige adipocytes have the thermogenic capacity to increase energy expenditure and thus are recognized to be used as a promising therapeutic avenue to counteract obesity and its associated metabolic syndromes [11, 12]. Cold exposure is a ubiquitous environmental stress which stimulates BAT activity and beige adipocyte formation (also known as WAT browning), and induces the expression of thermogenic UCP1 [13, 14]. Comprehensive studies have demonstrated distinct lipidomic or transcriptome profiles in brown and white adipose of mice and humans during activated thermogenesis [15–21]. Application of mass spectrometry and RNA sequencing (RNA-seq) revealed that 3-day cold stimulation may cause profound changes of the transcriptional landscape that regulates the lipid metabolism to fuel thermogenesis in BAT [15]. Acute 2-h cold exposure activates lipid metabolism in both BAT and the subcutaneous WAT depots, with the most striking change being observed in levels of diglyceride and monoglyceride in BAT [16]. A clear induction of *Ucp1*, *Elvol3*, *Cidea*, and many mitochondria-related genes was detected by transcriptome analysis in 7-day cold-treated iWAT [19]. Mice housed at 4 °C for 3 h, 3 days, 7 days, or 3 weeks displayed coordinately activated cardiolipin biogenesis in brown and white fat [20, 21].

The ability of adipose tissue to produce heat is a dynamic process that continues to increase with prolonged cold exposure, only reaching maximal capacity after several weeks [22]. Marcher et al. indicated that different experimental conditions (housing temperature for control mice or length of treatment) lead to different expression of the cold-regulated gene programs [15]. The top 250 genes in 3-day cold-induced BAT were different from that of the 1-day or 10-day cold-induced BAT [15]. Sustarsic et al. mapped the global BAT proteome throughout cold acclimation and revealed that the enrichment of lipid metabolism proteins far eclipsed that of proteins involved in all other metabolite pathways from 3 days to 3 weeks of cold exposure [21]. These previous data are informative to understand the dynamic processes of thermogenesis and suggest that 3-day cold exposure might be a turning point during the cold acclimation. However, the adaptive changes of lipidomic and transcriptomic profiles in the interrelation of lipid metabolism and regulatory pathways in iWAT upon 3-day cold exposure remain poorly characterized. Emerging evidence has suggested a pivotal role of diverse lipids in lifespan-extending interventions and has linked alterations in the lipidome to neurodegenerative disease, cancer, sepsis, wound healing, and pre-eclampsia [23, 24]. These findings suggest that lipid metabolism is directly linked to healthy, aging, and metabolic diseases [23, 24]. Although evidence indicates that cold-induced WAT browning ameliorates metabolic diseases and insulin resistance [11, 25, 26], defends against obesity during the aging process [27–29], and extends lifespan [30, 31], the interconnection between health/longevity and cold-activated lipidomic pathways in iWAT is still poorly understood.

In the present study, we applied mass spectrometry-based lipidomics combined with RNA sequencing (RNA-seq) to analyze the effect of short-term (3-day) cold exposure on the lipidome and gene expression of iWAT. We show that cold exposure leads to marked changes in the composition and content of different lipid classes, especially in the levels of glycerophospholipids and sphingolipids in iWAT. These changes were accompanied by activated fatty acid metabolism and elongation, as well as synthesis of TAG, sphingolipids, and glycerophospholipids. Moreover, our comprehensive datasets also revealed previously undescribed differences among the lipidomes of browning WAT, metabolic syndromes, and neurodegenerative disorders. Our databases constitute a valuable resource that may guide future investigations of the complex metabolic networks associated with the thermogenic program of WAT, and they help establish a link between the diverse species of lipids and physiologic metabolism in humans.

## Results

### Short-term cold exposure results in iWAT browning and body weight loss

To examine the short-term effects of cold stimulation on mice, we maintained mice at either room temperature (RT, 22 °C) or cold (4 °C) for 3 days and closely examined the body weight gain, adipose tissue weight, and food intake. We found that cold exposure resulted in body weight loss, which was mainly caused by the loss of adipose tissue mass (Additional file 1: Figure S1a, b). The food intake of the cold-treated mice was higher than that of the control mice, suggesting enhanced energy expenditure during cold exposure (Additional file 1: Figure S1c). Hematoxylin-eosin (H&E) staining revealed an obvious decrease in the individual cell size of both BAT and iWAT from cold-treated mice compared with that of controls (Additional file 1: Figure S1d). Notably, iWAT contained numerous small multilocular lipid droplets after cold treatment (Additional file 1: Figure S1d). Consistently, cold exposure dramatically increased the expression of BAT marker genes, such as *Ucp1* and PR domain containing 16 (*Prdm16*), but it decreased the mRNA levels of WAT markers (e.g., *Leptin*; Additional file 1: Figure S1e, f) in iWAT. It is still controversial about the suitable housing temperature for mouse to provide the model for understanding the mechanism of human disease [32, 33]. The effect of cold treatment on mice under different experimental conditions has been investigated by several studies [15, 20, 34–37]. In this study, the control mice were housed at room temperature around 22 °C, which is consistent with most previous studies [15, 34, 35] and our previous study [38]. These data demonstrate that 3-day cold exposure induces browning of iWAT, increases energy expenditure, and decreases body weight and fat mass.

### Cold exposure alters the overall composition of lipid classes in iWAT

**Fig. 1:**
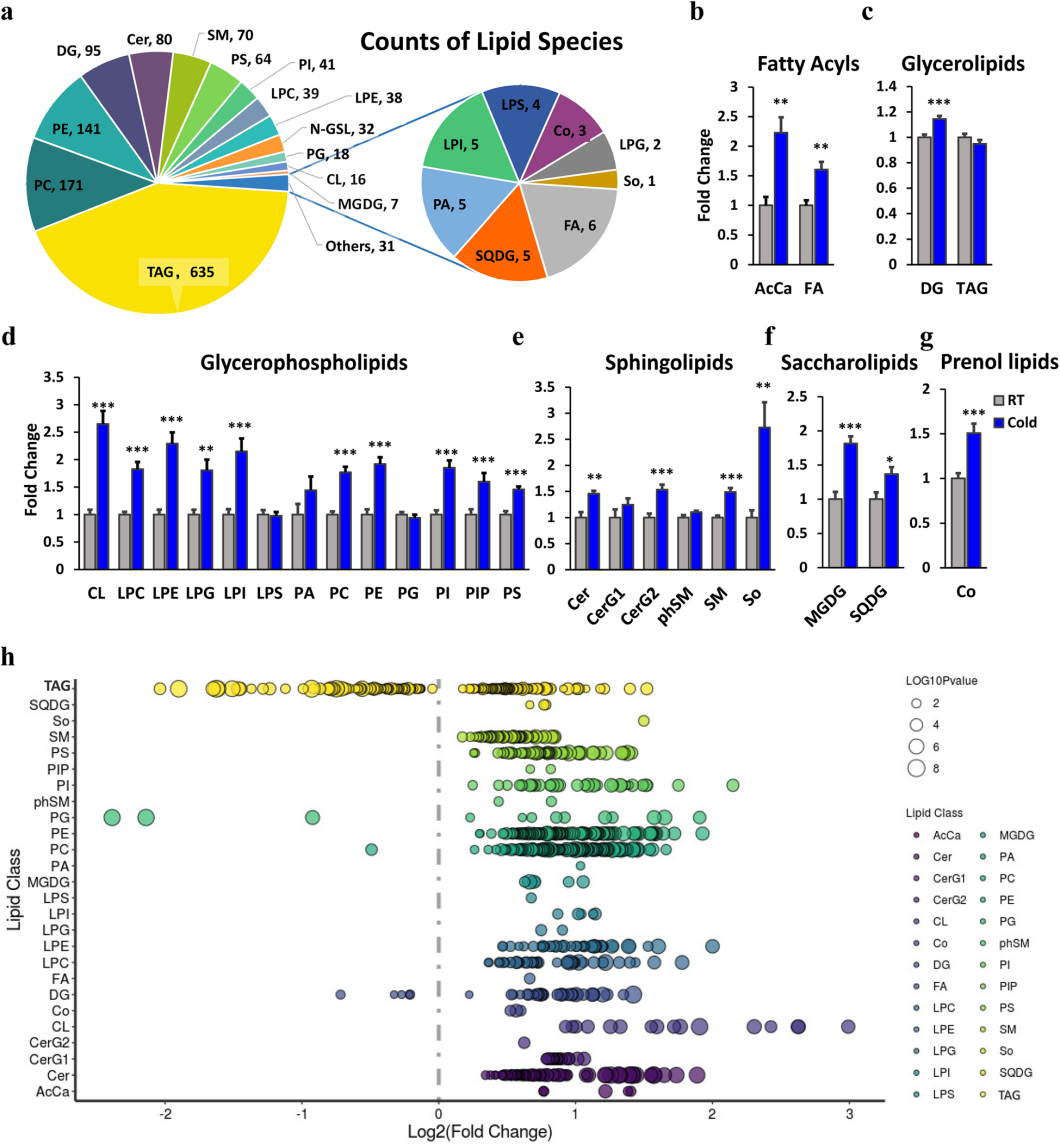
Changes of the overall lipid composition and distribution in iWAT in response to cold exposure. Lipidomic analysis of iWAT isolated from mice maintained at room temperature (RT, 22 °C) or cold (4 °C) for 3 days. Lipids were extracted and analyzed as described in the “Methods” section. **a** Distribution of lipid classes that were considered for subsequent analysis in all of the samples detected by LC-MS/MS. **b–g** The intensity fold change of fatty acyls (**b**), glycerolipids (**c**), glycerophospholipids (**d**), sphingolipids (**e**), saccharolipids (**f**), and prenol lipids (**g**). Data are presented as means + SEM (*n* = 8). *P < 0.05, ***P < 0.001. **h** Log2 fold changes in lipid species in cold-treated versus RT mice and the corresponding significance values displayed as -log10 (*P* value). Each dot represents a lipid species, and the dot size indicates significance. Only lipids with *P* < 0.05 are displayed (*n* = 8)

To determine the changes of the overall lipid composition and distribution in iWAT in response to cold, we isolated iWAT from RT- and cold-treated mice and applied mass spectrometry-based lipidomic analysis (Additional file 2: Table S1). The detected lipid classes and their abbreviations are shown in Additional file 3: Figure S2a. We detected over 1473 different lipid species in iWAT, consisting of 635 TAGs, 171 phosphatidylcholines (PCs), 141 phosphatidylethanolamines (PEs), and other lipid classes (Fig. 1a). The principal component analysis (PCA) plot shows clearly distinguished clusters of RT- and COLD-treat iWAT (Additional file 3: Figure S2b). The orthogonal projections to latent structures discriminant analysis (OPLS-DA) plot shows a clear separation of two classes (RT vs. COLD; R^2^X = 0.438, R^2^Y = 0.979, *Q*^2^ = 0.859; Additional file 3: Figure S2b-d; Additional file 4: Table S2). From the OPLS-DA model, 70 features were selected that differentiated between the two groups with variables importance for the projection of VIP > 1.0 and *P* < 0.001 (Additional file 3: Figure S2e). Relative quantification results show that short-term cold exposure significantly increased the following contents: the acyl carnitine (ACCA) and fatty acid (FA) in fatty acyls (Fig. 1b); the diglyceride (DG) in glycerolipids (Fig. 1c); the cardiolipin (CL), lysophosphatidylcholine (LPC), lysophosphatidylethanolamine (LPE), lysophosphatidylglycerol (LPG), phosphatidylcholine (PC), phosphatidylethanolamine (PE), phosphatidylinositol (PI), phosphatidylinositol (PIP), and phosphatidylserine (PS) in glycerophospholipids (Fig. 1d); the ceramiders (CER), simple gle series (CerG2), and sphingomyelin (SM) in sphingolipds (Fig. 1e); the monogalactosyldiacylglycerol (MGDG) and sulfoquinovosyldiacylglycerol (SQDG) in saccharolipids (Fig. 1f); and the coenzyme (CO) in prenol lipids (Fig. 1g). To further probe individual lipid species that were regulated by cold, we visualized all of the significantly changed lipid species using a bubble map. Using a *P* value of 0.05 as a cutoff, a total of 864 species were significantly changed in iWAT from cold-challenged animals (Fig. 1h). These findings suggest that cold exposure induces considerable alterations in the composition and content of lipid species.

### Cold exposure changes the expression of genes in lipid-metabolic pathways

**Fig. 2:**
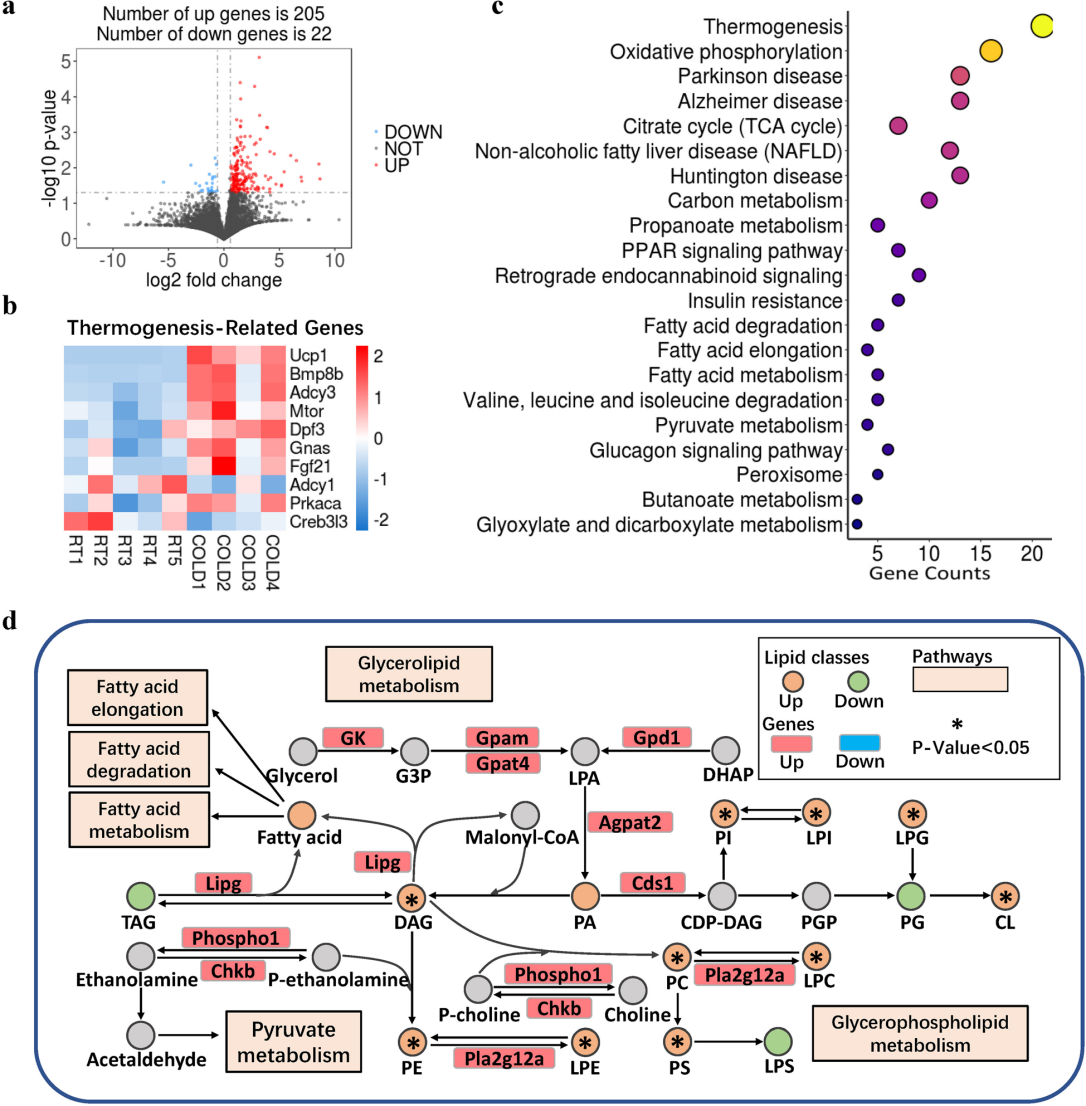
Short-term cold exposure induces gene programs involved in lipid metabolism. Mice were exposed to cold (4 °C, *n* = 4) or kept at room temperature (RT, *n* = 5) for 3 days. iWAT was isolated, and RNA was purified for RNA-seq. **a** Log2 fold changes in exons of RNA-seq gene bodies in cold-treated versus RT mice and the corresponding significance values displayed as log10 (*P* value). The transverse and vertical dotted lines indicate the cutoff value for differential expression (*P* < 0.05 & Abs (Log2 fold changes) > 0.585). In total, 205 and 22 genes were identified that had induced (red) or repressed (blue) expression levels by cold exposure. **b** Heatmap of relative expression of selected thermogenesis-regulated genes from the RNA-seq dataset. Only genes with *P* < 0.05 are displayed. **c** Gene Ontology (GO) enrichment analysis. **c** Functional enrichment analyses using Kyoto Encyclopedia of Genes and Genomes (KEGG) pathways. The triangle size indicates significance and corresponding significance values displayed as log10 (*P* value). **d** Selected glycerolipid and glycerophospholipid metabolic reactions from KEGG, with indications of quantified lipid classes and acyl chains (circles) and genes (rectangles) significantly regulated in iWAT by short-term cold exposure. Colors indicate increased (red) or decreased (blue) expression of genes (encoding proteins that catalyze the indicated conversions) upon cold exposure or increased (green), decreased (yellow), and undetected (gray) levels of the total concentration of the lipid classes

To explore how the iWAT lipidome is altered upon cold exposure, we applied RNA-seq to map the transcriptional changes and lipid-metabolic pathways in iWAT in response to short-term cold exposure (Additional file 5: Table S3). We found a total of 227 differentially expressed genes, of which 205 were increased and 22 were decreased by 3-day cold exposure (Fig. 2a). Consistent with the above results (Additional file 1: Figure S1), RNA-seq analysis showed that the expressions of the thermogenesis-related genes and BAT-selective genes, including *Ucp1*, *Prdm16*, *Cidea*, and iodothyronine deiodinase 2 (*Dio2*), were significantly upregulated (Fig. 2b; Additional file 6: Figure S3a). By contrast, the expressions of WAT-selective genes, including angiotensinogen (*Agt*), resistin (*Retn*), and tripartite motif containing 14 (*Trim14*), were significantly decreased (Additional file 6: Figure S3a). Cold exposure stimulates thermogenesis by increasing mitochondrial content and metabolic rate [13]. Notably, we also found a significant increase of peroxisome proliferator-activated receptor-gamma coactivator-1 alpha (PGC-1α) expression in iWAT, which is the key regulator of mitochondrial biogenesis (Additional file 6: Figure S3a). Gene ontology (GO) enrichment analysis of the genes induced by cold stimulation revealed pronounced changes in the mitochondrial membrane (Fig. 2c; Additional file 7: Table S4). GO enrichment analysis also showed that cold-induced genes were enriched in regulation of fatty acid oxidation, lipid oxidation, and fatty acid metabolic processes, and sequestering of TAG (Fig. 2c, Additional file 7: Table S4). Functional enrichment analyses using the Kyoto Encyclopedia of Genes and Genomes (KEGG) pathways [39, 40] revealed a significant enrichment of several major metabolic pathways (Fig. 2d; Additional file 8: Table S5). Genes IN thermogenesis-related pathways, including PPAR signaling, oxidative phosphorylation, and the TCA cycle, also showed dramatic changes in iWAT in response to cold (Fig. 2d). Pathways associated with fatty acid elongation, fatty acid degradation, and fatty acid metabolism had extensive changes in cold-induced iWAT (Fig. 2 d). The transcripts per million (TPM) analysis showed that fatty acid oxidation- and elongation-related genes were increased in iWAT upon cold exposure (Additional file 6: Figure S3b, c). In addition, the genes and pathways involved in glycerolipid and glycerphospholipid metabolism were also affected in cold-induced iWAT (Fig. 2e; Additional file 6: Figure S3d, e). Consistently, qPCR results confirmed the effects of cold exposure on the expression of genes involved in glycerophospholipid metabolism, glycerolipid metabolism, fatty acid elongation, β-oxidation, and sphingolipid metabolism in iWAT (Additional file 9: Figure S4). Moreover, cold exposure altered pathways related to three neurodegenerative diseases (Huntington’s disease, Parkinson’s disease, and Alzheimer’s disease) and insulin resistance (Fig. 2c). In addition, cardiomyopathy-related pathways (hypertrophic cardiomyopathy and dilated cardiomyopathy) significantly changed during cold exposure (Fig. 2c). These results suggest that cold exposure altered the expression of genes involved in thermogenesis, mitochondrial biogenesis, and lipid and fatty acid metabolic pathways.

Cold exposure caused extensive changes of glycerolipid and glyphospholipid pathways in BAT [15]. We found that the contents of glycerolipid and glycerphospholipid classes were significantly changed in iWAT in response to cold (Fig. 1c, d). To reveal the signaling pathways involved in glycerolipid and glycerphospholipid metabolism, we conducted conjoint analysis of lipidomics and transcriptomics. We provide an overview of selected glycerophospholipid metabolic-related genes from KEGG (Fig. 2d). The significantly increased genes that play a critical role in initial steps of glycerolipid and glycerophospholipid synthesis may partly explain the robust elevation of glycerolipid and glycerophospholipid contents.

### Cold exposure regulates the length of fatty-acyl chains associated with TAG

**Fig. 3:**
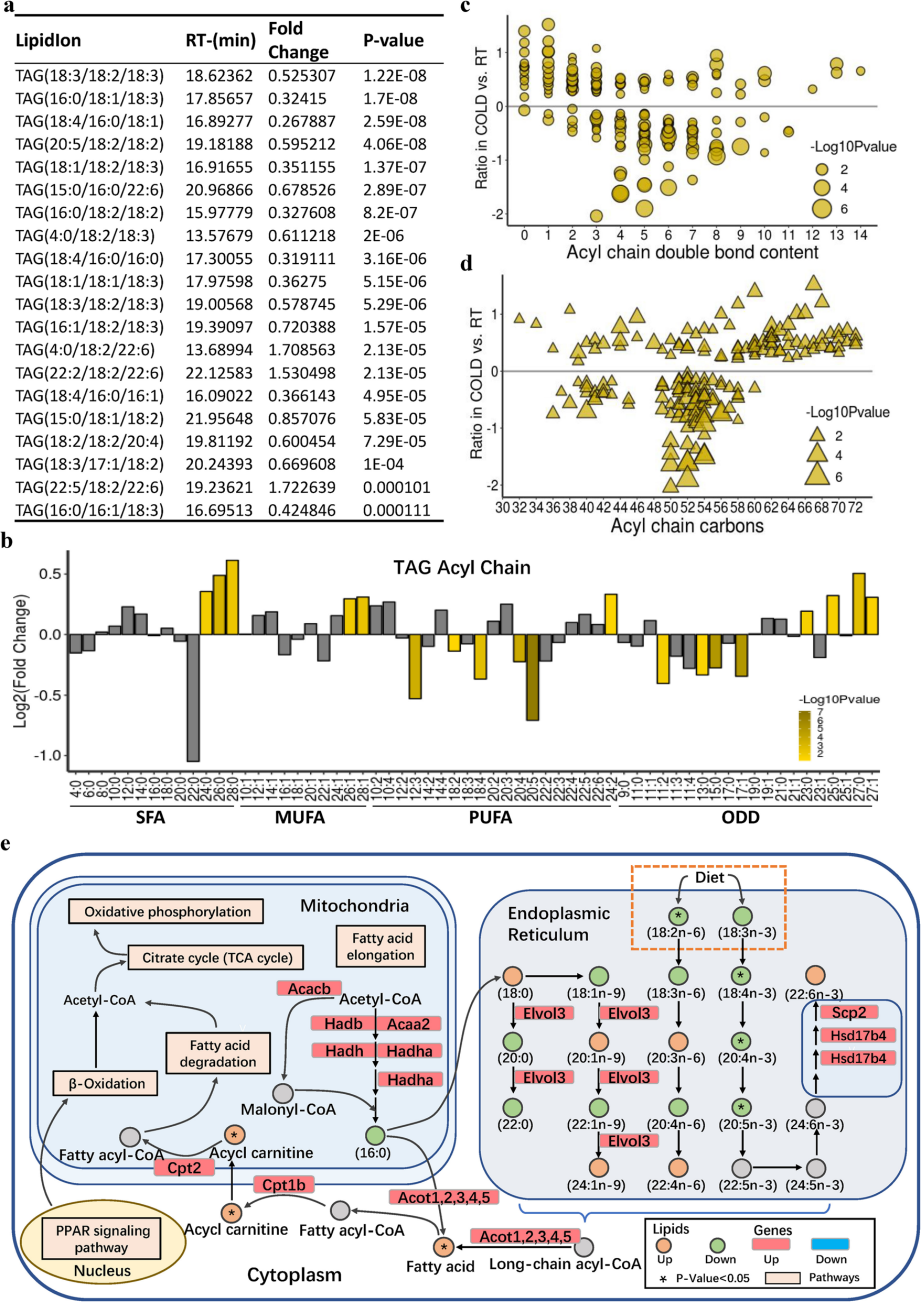
Cold-mediated changes in TAG composition and fatty-acyl chains in iWAT. **a** The top 20 TAGs according to the *P* value, detected in iWAT isolated from RT- and cold-treated mice (*n* = 8). **b** The total intensity fold changes of individual fatty-acyl chains associated with TAG sorted by degree of saturation. SAF, saturated fatty acyls; MUFA, monounsaturated fatty acyls; PUFA, polyunsaturated fatty acyls containing two or three to six double bonds; ODD, odd-numbered fatty acyls. The transparency of each bar is proportional to the significance values, which are displayed as -log10 (*P* value). The gray bars indicate those with *P* > 0.05. **c**, **d** The TAG pattern in cold-treated cases versus that in controls. Each dot or triangle represents a distinct TAG, organized along the *x* axis based on total acyl-chain carbon number (**c**) or double-bond content (**d**). The size of each dot or triangle is proportional to the significance values, which are displayed as -log10 (*P* value). Only lipids with *P* < 0.05 are displayed. **e** Selected fatty acid metabolic reactions from KEGG, with indications of quantified lipid classes and fatty-acyl chains (circles) and genes (rectangles) significantly regulated in iWAT by short-term cold exposure. For other abbreviations, see Fig. 2

Our RNA-seq results show that cold exposure altered the pathways associated with elongation and metabolism of fatty acids, which make up the bulk of TAGs (Fig. 2d). We next analyzed the individual fatty-acyl-chain composition associated with TAG, which reflects the major fatty acids that make up fat depots. We ranked TAG lipids according to the *P* values, compared cold and RT conditions, and examined the top 20 species individually. Among them, 17 species were significantly decreased and 3 species were significantly increased in iWAT by cold exposure (Fig. 3a). We found that most of the short-chain fatty-acyl chains were not changed by cold exposure (Fig. 3b). Notably, significant increases in the concentration of very-long saturated fatty-acyl chains (SFA; C24:0, C26:0, C28:0), monounsaturated fatty-acyl chains (MUFA; C26:1, C28:1), and polyunsaturated fatty-acyl chains (PUFA; C24:2) were found in the TAG pool of iWAT upon cold exposure (Fig. 3b). The concentrations of long polyunsaturated fatty-acyl chains (C12:3, C18:2, C18:4, C20:5) decreased in iWAT due to exposure to cold (Fig. 3b). In addition, we found that odd-numbered fatty-acyl chains (i.e., C15:0 and C17:1), which are generated by metabolism or absorbed from diet, were dramatically decreased in iWAT from cold-treated mice compared with those of mice exposed to RT (Fig. 3b). The long-chain odd-numbered fatty-acyl chains (i.e., C25:0, C27:0, and C27:1) were increased in iWAT after cold exposure (Fig. 3b). Moreover, we analyzed the total percentage of SFA, MUFA, and PUFA associated with TAG acyl chains, which make up the majority of lipids. Cold exposure decreased PUFA percentage, without affecting SFA or MUFA percentage in iWAT in response to cold (Additional file 10: Figure S5a). Higher MUFA/PUFA ratios have been observed in long-lived worms and in the daughters of long-lived humans, suggesting higher PUFA levels could be detrimental [41, 42]. The MUFA/PUFA ratio was increased in cold-treated iWAT compared with that in controls (Additional file 10: Figure S5b).

It has been reported that TAG species with lower carbon numbers and double-bond content have been associated with increased cardiovascular disease (CVD) [43]. TAG species with higher carbon numbers and double-bond content have been associated with a decreased risk of type II diabetes (T2D) [44, 45]. Interestingly, cold-induced iWAT displayed markedly elevated levels of TAG species with relatively lower double-bond content and higher acyl-chain carbon numbers (> 60 carbons) (Fig. 3c, d), suggesting that cold exposure improved TAG species that may have beneficial effects in treating T2D.

In addition, we found that cold exposure activated fatty acid elongation pathway and dramatically induced the expression of elongation of very long-chain fatty acids protein 3 (*ELOVL3*; Fig. 3e), which elongated both saturated and unsaturated C16-C22 acyl-CoAs with the highest activities toward C18-CoAs [46–48]. Pearson correlation analysis showed a significant positive correlation of the ratios of C18:2 verse C18:3 (Pearson *r* = 1), as well as C20:4 verse C20:5 (Pearson *r* = 0.9) (Additional file 10: Figure S5c). These findings demonstrate that the composition of fatty-acyl chains associated with TAG species in iWAT was markedly changed upon cold exposure, which was a result of increased expression of genes encoding enzymes involved in TAG synthesis and fatty acid elongation.

### Short-term cold exposure changes the composition of glycerophospholipids

**Fig. 4:**
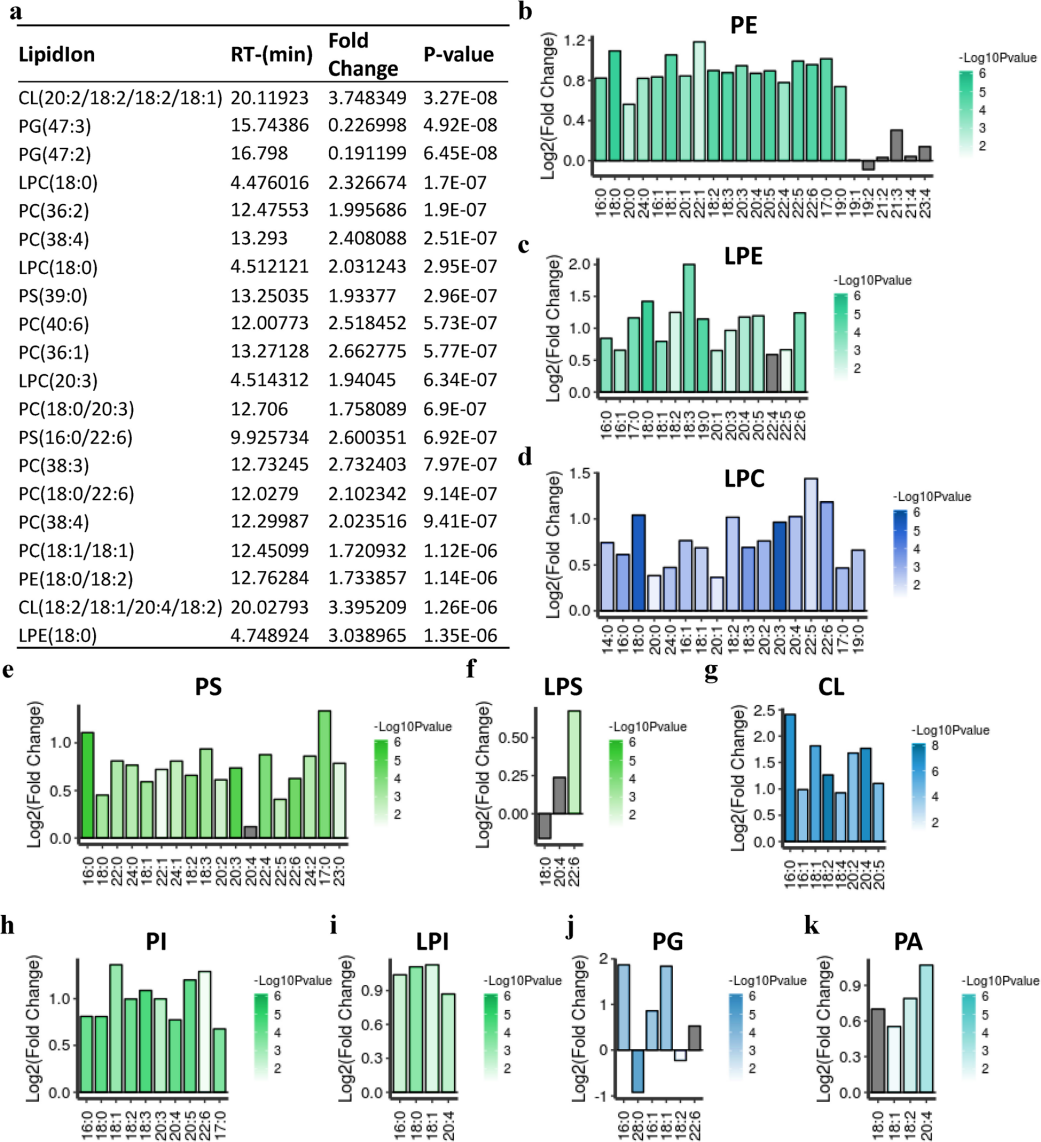
Short-term cold exposure changes the composition of glycerophospholipids. **a** The top 20 GPLs according to the *P* value, detected in iWAT from RT- and cold-treated mice (*n* = 8). **b–k** The intensity of individual fatty-acyl chains associated with different glycerophospholipid classes. The transparency of each bar is proportional to the significance values, which are displayed as -log10 (*P* value). The gray bars indicate those with *P* > 0.05. *P < 0.05, ***P < 0.001

Glycerophospholipids are major components of cellular membranes that regulate membrane fluidity, dynamics, and homeostasis [49]. We found that cold exposure increased the overall abundance of the different glycerophospholipid classes in iWAT (Fig. 1d). We next analyzed how the composition of the different glycerophospholipid classes is affected by cold exposure. We ranked lipids according to their *P* values, compared cold and room temperature conditions, and examined the top 20 species individually. Among them, 18 lipid species (including 9 PC lipids, 3 LPC lipids, 2 CL and PS lipids, and 1 PE and LPE lipid) were significantly increased and 2 lipid species (phosphatidylglycerol, PG) were significantly decreased in iWAT in response to cold (Fig. 4a). CLs are synthesized by coupling cytidine diphosphate-diacylglycerol (CDP-DAG) with PG in the mitochondrial inner membrane [21]. Sustarsic et al. indicated that CLs and PGs were robustly induced in subcutaneous WAT (scWAT) upon cold exposure for 3 weeks [21]. Inconsistent with the significantly increased level of CLs, the total level of PGs was not affected in 3-day cold-induced iWAT, while the levels of two PG species contained in the top 20 lipid species were decreased (Figs. 1d and 4a). It has been reported that a set of docosahexaenoic-acid-enriched phospholipids (DHA-PLs) showed the highest specificity for BAT [50]. In the current study, we found that glycerophospholipid species with DHA (C22:6) were contained in two of the top 20 lipid species, namely PS (16:0/22:6) and PC (18:0/22:6) (Fig. 4a). When analyzing the total pool of fatty-acyl chains associated with different glycerophospholipid classes, we found that most of the fatty-acyl chains were significantly increased (Fig. 4b–k). There exists much data on the therapeutic potential of arachidonic acid (20:4n-6; AA), epoxygenases, eicosapentaenoic acid (20:5n-3; EPA), and docosahexaenoic acid (22:6n-3; DHA) in protecting against cardiac arrhythmia, triglyceride levels, inflammation, and neurodegenerative disorders [51]. In this work, we found that C20:4 was significantly increased in PE, LPC, PS, LPS, CL, PI, LPI, and PA (Fig. 4b, d–i, k); C20:5 was significantly increased in PE, LPE, CL, and PI (Fig. 4b, c, g, h); and C22:6 was significantly increased in PE, LPE, LPC, PS, LPS, PI, and PG (Fig. 4b–f, h, j). Sustarsic et al. reported that there was a significant increase in phosphatidylglycerol species containing fatty acid tails 18:2, 18:1, 16:1, and 16:0, which serves as a substrate of cardiolipin synthesize in BAT and scWAT form mice housed in cold for 7 days. Here we found that fatty acid tails 16:0, 16:1, 18:1, 18:2, 20:2, 20:4, and 20:5 in CLs were significantly increased by 3-day cold exposure. In conclusion, cold exposure induced the accumulation of glycerophospholipid species (GPLs), especially DHA (C22:6)-GPLs, EPA (C20:5)-GPLs, and AA (C20:4)-GPLs.

### Cold exposure selectively increases the overall abundance of sphingolipid classes in iWAT

**Fig. 5:**
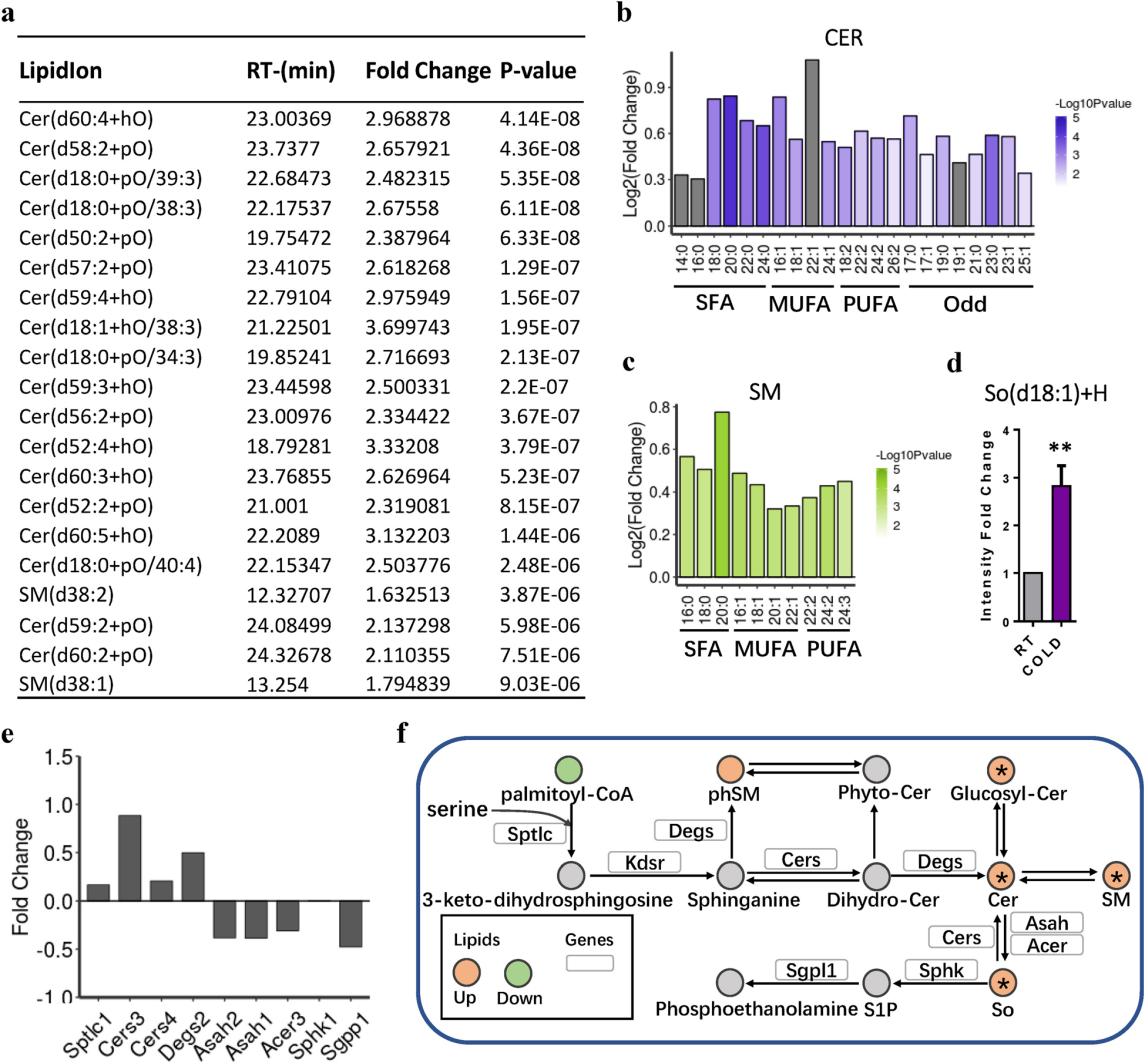
Cold exposure changes the composition of sphingolipids. **a** The top 20 sphingolipids according to the *P* value, detected in iWAT from RT- and cold-treated mice (*n* = 8). **b**, **c** The total intensity fold changes of individual fatty-acyl chains associated with ceramides (CER) and sphingomyelin (SM) sorted by degree of saturation. SAF, saturated fatty acyls; MUFA, monounsaturated fatty acyls; PUFA, polyunsaturated fatty acyls containing two or three to six double bonds; ODD, odd-numbered fatty acyls. The transparency of each bar is proportional to the significance values, which are displayed as -log10 (*P* value). The gray bars indicate those with *P* > 0.05. **d** The total intensity fold changes of individual fatty-acyl chain associated with ceramides So. **e** RNA-seq reveals relatively expression of genes involved in sphingomyelin biogenesis and breakdown. Data are presented as means + SEM (*n* = 4–5). **f** Selected sphingolipid metabolic reactions from KEGG, with indications of quantified lipid classes and acyl chains (circles) and genes (rectangles) significantly regulated in iWAT by short-term cold exposure. For other abbreviations, see Fig. 2

Compared with glycerolipids and glycerophospholipids, sphingolipids (e.g., ceramides, sphingomyelins, and sphingosine) are very low in abundance in the body [52]. However, sphingolipids are a diverse class of essential cellular lipids that function as structural membrane components [53], and many sphingolipid species have been implicated as critical metabolites linking obesity to T2D, cardiovascular disease, and metabolic disorders [52, 54, 55]. We found that cold exposure increased the level of numerous sphingolipid species in iWAT (Fig. 1e). We next examined the effect of cold exposure on the composition of the different sphingolipid classes. We ranked SM and Cer lipids according to their *P* values and compared cold and RT conditions, and the top 20 species, including 2 species of SM and 18 species of Cer, were significantly increased (Fig. 5a). When analyzing the total pool of fatty-acyl chains associated with SM and Cer, we detected a marked elevation in C20:0 in both Cer and SM (Fig. 5b, c). The level of C24:0 and C23:0 in Cer also showed a significant increase (Fig. 5b). Interestingly, the odd-numbered fatty-acyl chains associated with Cer were also markedly increased (Fig. 5b). The level of C17:0, which was decreased in TAGs and significantly increased in glycerophospholipids, was significantly increased in Cer (Fig. 5b). Moreover, the levels of very long odd-numbered fatty-acyl chains (C23:0, C23:1, C25:0) in Cer were significantly increased (Fig. 5b). The only kind of sphingosines, detected in this work, So (d18:1), was significantly increased in iWAT due to cold exposure (Fig. 5d).

A previous study indicated that de novo sphingolipid biosynthesis is required for adipocyte survival and metabolic function [53]. The de novo biosynthesis pathway of sphingolipids is complex and is regulated by a series of enzymes (Fig. 5e). qPCR analysis also revealed that cold exposure increased mRNA expression of serine palmitoyltransferase long chain base bubunit 1 (*Sptlc1*), the first enzyme in the sphingolipid biosynthesis cascade, and ceramide synthase 1 (*Cers1*), which catalyzes subsequent enzymatic reactions (Additional file 9: Figure S4e). The RNA-seq results also revealed that de novo sphingolipid-synthesis-related genes (*Sptlc1*, *Cers4*, and *Degs2*) tended to increase and sphingolipid-breakdown-related genes (*Asah1*, *Asah2*, *Acer3*, *Sphk1*, and *Sgpp1*) tended to decrease (Fig. 5e, f). These results indicate that cold exposure activates de novo sphingolipid synthesis and induces accumulation of sphingolipid species in iWAT.

### Comparison of genetic and lipidic programs in cold-treated and disease models

**Fig. 6:**
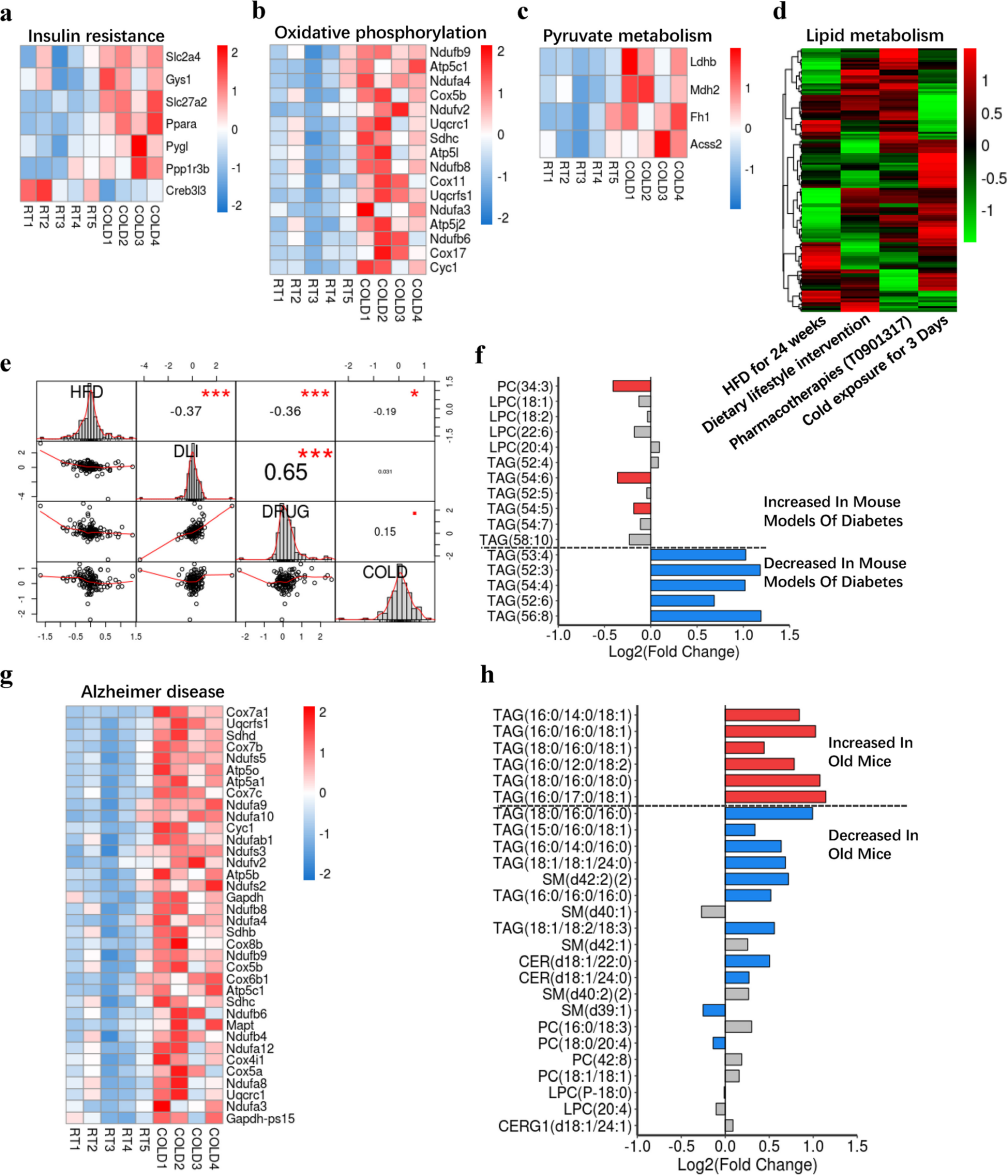
Comparison of genetic and lipidic programs in cold-treated and disease models. **a–c** KEGG results of the cold-induced enrichment of genes involve in insulin resistance (**a**), oxidative phosphorylation (**b**), and pyruvate metabolism (**c**). **d** Fold changes of the lipid metabolism-related genes in different models. **e** Correlation matrix for all included studies based on Pearson’s correlation coefficient for the subset of genes with cold-induced expression described in **d**. **f** Log2 fold changes of specific lipids (considered as plasma biomarkers in diabetics) in cold-induced iWAT. **g** Scaled expression values for five control (RT) and four cold-treated (4 °C) mice for genes with significant enriched KEGG pathway associated with Alzheimer disease. **h** Log2 fold changes for lipid species (significantly changed in old mouse models) in cold-induced iWAT

As previously mentioned, KEGG enrichment analysis of genes with the most pronounced cold-induced expression revealed a significant enrichment in metabolic pathways, including insulin resistance, oxidative phosphorylation, and pyruvate metabolism (Fig. 2d). Insulin resistance is the major contributor to the etiology and pathogenesis of T2D mellitus [56]. Oxidative phosphorylation is the primary source of metabolic energy [57] and is a potential target in the treatment and prevention of obesity-related metabolic disorders [58]. Pyruvate is a keystone molecule critical for numerous aspects of eukaryotic metabolism and drives ATP production by oxidative phosphorylation and multiple biosynthetic pathways in mitochondria [59]. Aberrant pyruvate metabolism plays an especially prominent role in neurodegeneration, cancer, and heart failure [59]. Heatmap results showed a significant enrichment of gene expression disruption in pathways relevant in insulin resistance (Fig. 6a), oxidative phosphorylation (Fig. 6b), and pyruvate metabolism (Fig. 6c). These significant alterations in the expression of metabolic pathway-related genes suggest a potential relevance between cold exposure and preventing obesity-related metabolic disorders. Moreover, we also compared the fold changes in the expression of lipid metabolism-related genes in our current study and previous studies, including high-fat diet (HFD)-induced obesity [60], dietary lifestyle interventions [61], and anti-diabetic drugs and cold exposure [61] (Fig. 6d; Additional file 11: Table S6). It has been reported that dietary lifestyle interventions and anti-diabetic drugs can improve the symptoms of diabetes [61]. Consistently, there is a dramatically negative correlation in the expression of lipid metabolism-related genes between dietary lifestyle interventions and HFD-induced obesity, as well as between anti-diabetic drugs and HFD-induced obesity (Fig. 6d, e). We also found a significantly negative correlation in the expression of lipid metabolism-related genes between HFD-induced obesity and cold exposure, suggesting that cold exposure might be a potential therapeutic strategy for obesity and diabetes (Fig. 6e). There is a moderate positive correlation between cold exposure and dietary lifestyle interventions, as well as between cold exposure and anti-diabetic drugs, suggesting that cold exposure, dietary lifestyle interventions, and anti-diabetic drugs affect different pathways to improve lipid metabolism (Fig. 6e).

Moreover, a transcript-lipidomic correlation network was built to screen the significantly changed genes and lipids located at key nodes in the network of regulation and biosynthesis of lipids during cold exposure (Additional file 12: Table S7; Additional file 13: Figure S6a). The top 20 genes and top 20 lipids with the greatest numbers of correlated nodes are shown in Additional file 13: Figure S6b-c. Carbon metabolism (DLAT, sdhD), the PI3K-Akt signaling pathway (Fgf23), BMP8b, and Fndc5 play critical roles in this process (Additional file 13: Figure S6b). TAGs and PEs may be potential targets for energy homeostasis during cold acclimation (Additional file 13: Figure S6c).

Obesity concentrations are accompanied by metabolic side effects, such as high plasma-TAG concentrations. WAT is an important regulator of plasma TAG. Exposure to 4 °C reduces plasma-TAG concentrations [62]. We therefore aimed to study the interaction between diabetes and 3 days of exposure to 4 °C on the concentrations of lipid species, respectively, in plasma and iWAT. We analyzed the alteration of specific lipids, which are considered to be biomarkers in diabetics (Fig. 6f). Most of these biomarkers had opposite trends in cold-induced iWAT, such as PC (34:3), TAG (54:6), TAG (54:5), TAG (53:4), TAG (52:3), TAG (54:4), TAG (52:6), and TAG (56:8) (Fig. 6f). Recent clinical and basic studies have proposed the term “Type-3-Diabetes” or “brain diabetes” for Alzheimer’s disease (AD) because of the shared molecular and cellular features and bidirectional interactions between diabetes and AD [63–65]. Various clinic and basic biological studies have indicated that insulin signaling also plays a crucial role in AD [66–68]. Our KEGG analysis results revealed that the cold-induced enrichment of genes was related to AD regulation pathways (Fig. 2d). Most of the elevated genes were related to mitochondrial biogenesis and function (Fig. 6g), suggesting a potential protecting effect of cold exposure on AD. It is well known that aging is the greatest risk factor for Alzheimer’s disease. We analyzed the alteration of specific lipids, which were significantly decreased in older mice. We found that most of the lipids that were decreased in old mice were increased in iWAT in response to cold (Fig. 6h). In addition, we also compared the lipidic programs in iWAT from cold-treated models and the plasma from insulin-resistant and obese/overweight patients [44] (Additional file 14: Figure S7a-e). Most of these marker lipid species were increased in iWAT from 3-day-treated mice (Additional file 14: Figure S7a-d). Interestingly, the lipid profiles in cold-induced iWAT were mostly opposite to the changes of the specific lipids found in obese/overweight patients (Additional file 14: Figure S7e). Taken together, most of the cold-induced changes in expression of lipid metabolism-related genes and levels of specific lipid species were opposite to those in models of diabetes, Alzheimer disease, and aging, suggesting that lipid metabolism dysfunction in these metabolic disorders might be partly reversed by cold exposure.

## Discussion

The induction of iWAT thermogenic capacity by cold exposure is a multistep process that involves a complex interplay between transcriptional and metabolic signaling pathways. In this study, we applied RNA-seq and mass spectrometry-based lipidomics to extend our understanding of the alteration of lipid metabolism and its pathways in iWAT in response to short-term (3-day) cold exposure. Our results show that cold exposure induced significant changes in the overall composition of lipid classes, the length of acyl chains associated with TAG and the levels of glycerophospholipids and sphingolipids in iWAT. RNA-seq results indicate that a cold challenge induced gene programs involved in regulation of thermogenesis, fatty acid metabolism, TAG and glycerophospholipid synthesis, insulin resistance, and several disease-related pathways. Moreover, the global changes in the lipidome that occurred during cold adaption in iWAT are functionally linked to alterations of metabolic pathways. In addition, we compared the cold-altered lipids and gene profiles in iWAT with those of metabolic diseases and found that several cold-induced-specific lipids and pathways may have beneficial effects on health and lifespan. Our results have revealed cold-induced lipid dynamics and gene programs in iWAT and provide lipodomic and transcriptional signatures of iWAT in response to cold.

Using untargeted lipidomics, we found that lipid classes are remodeled selectively by 3-day cold adaption in iWAT, especially in term of the contents of glycerolipids and glyphospholipids. Unlikely, the concentrations of major lipid classes in BAT are only affected marginally by 3-day cold exposure [15]. A longer time (7-day) of cold stress also did not alter the total amount of most of the lipid classes in BAT and WAT [20]. Other than the absolute quantification applied in previous studies [15, 20], the relative quantification method we used in this study might exaggerate the differences of the lipid class contents in iWAT response to cold exposure. The individual lipid species that were significantly regulated by cold were visualized in the bubble chart. An enrichment of changes to TAG lipids can be observed in iWAT. Consistently, there were numerous dramatic changes in the composition of the TAG species in BAT [15]. TAG species also contribute the most to the number of lipid species significantly regulated by 7-day cold treatment in both BAT and WAT [20]. While TAG species are major substrates for lipid oxidation, almost half of significantly changed TAG species are increased by 3-day cold exposure in iWAT. However, longer time (7-day) cold stress decreased most of significantly changed TAG species in iWAT [20]. The different distribution pattern of significantly changed TAG species induced by 3-day and 7-day cold exposure was similar in BAT [15, 20]. Our previous study indicated that WAT mass was greatly reduced after 7-day cold treatment [38]. We hypothesize that cold acclimation for 7 days burned too much fat, leading to the significantly changed TAG species in both BAT and WAT [15, 20]. Thus, the lipidomic alterations induced by short time (3-day) cold stress might reflect the lipid metabolism regulation during early cold acclimation. Otherwise, transcriptome analyses and RT-qPCR reveled significant alterations of several genes (*Gpd1*, *Gpam*, and *Lipg*) involved in glyphospholipid and glycerolipid pathways between cold exposure and RT. *Gpd1* encodes a member of the NAD-dependent glycerol-3-phosphate dehydrogenase family, which catalyzes the reversible conversion of dihydroxyacetone phosphate (DHAP) and reduces nicotine adenine dinucleotide (NADH) to glycerol-3-phosphate (G3P) and NAD+ [69]. In addition to its role in triglyceride metabolism [70], GPAM esterifies long-chain fatty-acyl coenzyme-A to G3P to produce LPA, which is further metabolized to PA [71]. The cold-induced initial steps of glyphospholipid synthesis in iWAT might be responsible for the dramatically increased level of glyphospholipid content. The changes of TAG, DAG, and fatty acid contents might be a result of the elevated expression of *Lipg*, which encodes endothelial lipase to hydrolyze TAGs and diglycerides [72].

We also found a significant increase of the CL, LPC, LPE, LPG, PC, and PE in glycerophospholipids in iWAT upon short-term cold exposure. Heat-generating BAT, with its high density of mitochondria, was reported to contain more phospholipids than energy-storing WAT, which is in accordance with their respective metabolic functions [50]. Phospholipid metabolism was activated by cold exposure in brown and beige adipocytes to support mitochondrial biogenesis [15, 20]. In this study, cold exposure altered the expression of genes involved in thermogenesis, mitochondrial biogenesis, and lipid and fatty acid metabolic pathways. The activated phospholipid biogenesis and dramatically elevated content of phospholipids in iWAT might be the consequence of increased mitochondrial content and improved mitochondrial function during cold adaption. Among these glycerophospholipids, CLs are the signature phospholipids of the inner mitochondrial membrane and are important for the stability and activity of mitochondrial-protein machineries [73–75]. We observed a significant difference in the total concentration of CLs and the most abundant cardiolipin species, CL (20:2/18:2/18:2/18:1) and CL(18:2/18:1/20:4/18:2) in iWAT response to cold exposure for 3 days. Sustarsic et al. revealed that cardiolipin biogenesis, which is essential for systemic energy homeostasis and insulin sensitivity, was induced by cold exposure in brown and beige fat [20, 21]. The mRNA level of *Crls1* was increased by 3-week cold exposure in both iBAT and scWAT [20, 21]. However, the mRNA level of *Crls1* was not affected by cold exposure for 3 days in iWAT in this study, as well as other cardiolipin de novo synthesis and remodeling pathway genes, like phosphatidate cytidylyltransferase 2 (*Cds2*), phosphatidylglycerophosphate synthase 1 (*Pgs1*), and protein tyrosine phosphatase mitochondrial 1 (*Ptpmt1*). The inconsistent results suggest that 3-day cold exposure might be too short to induce a significant alteration in cardiolipin biogenesis pathway. In addition, the housing temperature also regulates the lipid metabolism in mice. The housing condition of Sustarsic et al. was thermoneutrality (29 °C) and cold (5 °C) [21]. However, in our study, the housing temperature was 25 °C for RT and 4 °C for cold. Otherwise, the individual difference within groups in this study also partly exerts influence in the profile of cardiolipin biogenesis-related genes, as there was not enough sample size (*n* < 6) for transcriptome analysis in this study. We hope that future work based on the content of proteins or activity of enzymes involved in cardiolipin biogenesis will provide a greater understanding of the role of CLs in browning of iWAT response to cold exposure.

Our data demonstrate that short-term cold exposure led to enrichment of very long acyl chains associated with TAGs, glycerophospholipids, and sphingolipids in iWAT. Very long-chain fatty acids (VLCFAs) are important components of different classes of lipids in all organisms from bacteria to man [45]. The VLCFA level in BAT was not significantly affected by cold exposure, except for C22:0, which was markedly elevated [15]. We found that VLCFAs in TAGs, glycerophospholipids, and sphingolipids in iWAT from cold-treated mice showed more pronounced accumulations compared with those of other fatty acids, which suggests that these species (C24:0, C26:0, and C28:0) could be functionally involved during cold adaption. Moreover, our RNA-seq results revealed extensive changes in fatty acid metabolic pathways in iWAT in response to cold exposure. Combined with other findings [19, 48], we conclude that the activated fatty acid elongation pathway and the increased expression of Elovl3 induced by cold exposure may result in increased levels of very long fatty acids. Fatty acids are converted by beta-oxidation to acetyl-coenzyme (CoA) in the form of acyl-CoA molecules in mitochondria. The very long-chain acyl-CoA dehydrogenase (VLCAD) catalyzes the first step of mitochondrial beta-oxidation for VLCFAs. VLCAD-deficient patients display severe hypoglycemia, cardiomyopathy, deficient mitochondrial β-oxidation of LCFAs, and a high-energy demand in tissues and organs [76]. BAT from the VLCAD-deficient mice showed a similar gene expression pattern with that from cold-treated mice, including elevated levels of UCP1 and PPARα [76]. Fatty acid oxidation also occurs in peroxisomes when fatty acid chains are too long to be handled by the mitochondria. A previous study observed strong upregulation of peroxisomal β-oxidation in VLCAD(−/−) mice [77]. We found that short-term cold exposure induced a significant increase of *Ehhadh* and *Hadha*, two genes involved in the peroxisomal beta-oxidation pathway, which might serve as a compensatory mechanism to produce greater energy in response to cold exposure. We also found that ACADVL, the gene that encodes VLCAD, was dramatically increased in iWAT in response to cold exposure, which might be a consequence of the elevation of VLCFAs. We hypothesize that such dramatically increased VLCFAs are also conceivable in iWAT from VLCAD-deficient mice in response to cold exposure and that the disturbed mitochondrial capacity for VLCFA beta-oxidation in iWAT may contribute to the cold sensitivity in VLCAD-deficient individuals.

We observed a significant alteration of the odd-numbered fatty-acyl chains in iWAT in response to cold exposure. Recent studies have reported that odd-numbered fatty acids (ONFAs) are detected not only in human plasma and RBCs but also in liver, buccal, and adipose tissues [78]. Abundant studies have shown an inverse association between ONFA concentrations in human plasma phospholipids or RBCs and the risk of T2D [79–83] and cardiovascular disease [84–86]. In BAT upon cold exposure, TAG species containing odd-numbered fatty-acyl chains (i.e., C15:0 and C17:0) show the most dramatic relative increases, although these are minor TAG species [15]. However, TAG species containing odd-numbered fatty-acyl chains (i.e., C11:2, C13:0, C15:0, and C17:1) were significantly decreased in iWAT upon cold exposure. TAG species containing very long odd-numbered fatty-acyl chains (i.e., C23:0, C25:0, C27:0, and C27:1) showed remarkable elevations in their expressions. We hypothesize that cold-induced odd-numbered fatty acid changes might be biomarkers or metabolic regulators to regulate systematic homeostasis during cold adaption. Odd-numbered fatty acids can be synthesized endogenously by elongation of shorter-chain ONFAs or can be derived from degradation of VLCFAs. Otherwise, it has also been observed in cultured differentiating adipocytes that C15:0 may also be formed from hexadecanoic acid (16:0) after intermediate hydroxylation [87]. Odd-numbered fatty acids are less preferred substrates for β-oxidation-related enzymes than even-numbered fatty acids, and the final products are propionyl-CoA and acetyl-CoA [88]. Accumulating propionyl-CoA within the mitochondria can be transported to the cytoplasm and plasma as C3-acylcarnitine by the combined action of Cpt2 and CACT and provide an aplerotic intermediate for the TCA cycle when mitochondrial energy production is impaired. The observed decreased levels of C15:0 and C17:0 in TAG (Fig. 3b) in iWAT in response to cold exposure are possibly a result of lipolysis and fatty acid oxidation, which provides anaplerotic intermediates to the TCA cycle and improves mitochondrial metabolism, thereby producing more energy to defend against hypothermia during cold adaption. As serum pentadecanoic acid (15:0) is inversely associated with incident T2D and its underlying disorders [83], the cold-induced dramatically decreased level of C15:0 in TAG, which accounted for most of the proportion of total lipids in iWAT, might provide evidence that cold treatment is beneficial for T2D. Odd-numbered fatty acids also serve as a substrate for the synthesis of odd-numbered VLCFAs for glycosphingolipids in the brain and elsewhere [89]. The increased level of odd-numbered VLCFAs in Cer (Fig. 5b) is possibly synthesized by these medium-chain odd-numbered fatty acids. However, the details of the regulation of odd-numbered fatty acid metabolism and synthesis remain unclear.

Genetic correlation analysis of the plasma lipidome with T2D, prediabetes, and insulin resistance revealed six lipid species, which belong to the triacylglycerol class and contain palmitate at the first position and may be genetically correlated with the risk of diabetes, prediabetes, and insulin resistance [90]. In the current study, three of the top 20 significantly changed TAGs containing palmitate at the first position, TAG (16:0/18:1/18:3), TAG (16:0/18:2/18:2), and TAG (16:0/16:1/18:3), were significantly decreased by cold treatment, which suggests a potential protection capability of cold exposure against the lipid metabolic disorder in T2D. Individual fatty-acyl-chain composition associated with TAG was also influenced by cold exposure. Although most of the shorter-chain fatty-acyl chains were not affected by cold exposure, the increased fatty acids (C12:0, C14:0, C14:1, C19:1, and C20:1) and decreased fatty acids (C16:0, C20:0, and C 22:0), observed in the differentiated primary adipocytes [87], had the consistent tendencies induced by cold exposure in iWAT.

We also observed a significant increase of DHA (C22:6)-GPLs and EPA (C20:5)-GPLs in iWAT. In addition to its contribution to membrane fluidity, higher DHA intake and plasma levels have cognitive benefits [91, 92]. Tracer studies suggest that DHA-PLs more effectively target the brain than DHA-enriched triglyceride (DHA-TAG) [93]. The trend toward higher DHA-GPL levels in iWAT browning in this work appears reasonable as it is in line with a previous study in mice showing that DHA-PLs dominate the BAT-discriminatory phospholipids, compared with iWAT [50]. Both DHA-PC and DHA-PS significantly improved the metabolic disorders and cognitive deficits. EPA and DHA protect against multiple metabolic and neurologic disorders. Although DHA appears to be more effective for neuroinflammatory conditions, EPA also protects against multiple metabolic and neurologic disorders, especially depression.

Sphingolipids are ubiquitous components of eukaryotic membranes. Previous studies showed that numerous adipose sphingolipids are elevated in human obesity and diabetes and correlate with related complications [94–97]. Accumulating data indicate that inhibition of sphingolipids may be a useful strategy for the treatment and prevention of obesity and associated complications. However, Alexaki et al. demonstrated that de novo sphingolipid biosynthesis is required for adipocyte cell viability and normal metabolic function and that adipocyte-specific deletion of Sptlc1 resulted in a striking age-dependent loss of adipose tissue accompanied by evidence of adipocyte death and metabolic dysfunction [53]. Moreover, high blood Cer levels have been shown to predict cognitive impairment and Alzheimer’s disease [98], which is the most common neurodegenerative disease. Chaurasia et al. indicated that cold and β-adrenergic-induced thermogenesis decrease ceramides in WAT and adipocyte-specific Sptlc2 ablation induces thermogenesis, brown/beige adipocyte accumulation, and M2 macrophage recruitment and activation in subcutaneous adipose tissue [97]. However, sphingomyelin (SM) and ceramide (Cer) were unaffected by cold exposure in WAT and BAT [15, 20]. In the present study, lipidomics revealed a significant elevation of sphingomyelin and ceramide in iWAT in response to cold exposure. Consistently, sphingolipid biogenesis genes (Sptlc1, Cers4, and Degs2) tended to increase and sphingolipid-breakdown-related genes (Asah1, Asah2, Acer3, Sphk1, and Sgpp1) tended to decrease. Responding to hormonal and energetic cues, energy-rich fatty acids stored in adipose tissue were released to mitochondria and target tissues in need of energy during cold exposure. Accumulating evidence has indicated that free fatty acids, proinflammatory cytokines, oxidative stress, and hormones may activate ceramide synthesis [99, 100]. In this regard, the elevated sphingolipid in iWAT from cold-treated mice might be a secondary result of increased availability of free fatty acids induced by cold exposure.

It is generally accepted that mitochondrial function is compromised in several disorders, such as obesity, T2D, and aging [101, 102]. In addition, background insulin resistance is the major pathogenesis underlying metabolic syndrome and AD. Thermoregulatory deficits coincide with a rise in the incidence of metabolic syndrome and a set of neurodegenerative disorders in old age [103]. A previous study indicated that thermogenesis stimulation improved metabolic deficits and protected a mouse model of AD [104]. Increasing evidence has suggested that adipose tissue influences brain development, cognition, and the risk for neurodegenerative disorders throughout one’s lifespan [105]. In this context, it is likely that the peripheral-derived metabolic changes in iWAT triggered by cold exposure may also provide beneficial effects on T2D and neurodegenerative disorders. Our transcriptomic data revealed a comprehensive and valuable association between cold treatment and metabolic pathways, such as insulin signaling and PPAR pathways. Functional enrichment analyses of genes with pronounced cold-induced expression in the present study revealed a set of genes associated with neurodegenerative disorders. Several adipokines have been implicated in AD, such as FGF21, adiponectin, and resistin [105]. The gene that encodes FGF21 was significantly increased, while the others tended to decrease in response to cold exposure in iWAT. Given that lipids are involved in multiple metabolic pathways, efforts to understand the pathogenesis of metabolic syndrome and neurogenic diseases are increasingly focused on disordered lipid metabolism. Several studies have reported the lipidomic profile of plasma or muscle from obesity, T2D, and aging subjects [41, 106, 107]. Our data indicated that most of the significantly changed lipid species in plasma or muscle from obesity, insulin resistance, and T2D also have a marked alteration in iWAT from cold-treated mice. The potential signaling roles of other individual lipids altered in these disorders remain to be explored. We hypothesize that enhanced thermogenesis through cold exposure could exert benefits on metabolic endpoints during aging. A better understanding of lipid metabolism changes triggered by cold treatment could reveal new clues in metabolic syndrome and neurodegenerative disorders. A great deal of lipidomic data has been accumulated regarding alterations of tissue and plasma lipids in cold exposure conditions. The major challenge for lipidomic research is to unravel the molecular mechanisms of how lipid changes in tissue and plasma are associated to metabolic pathways and to correlate specific lipid species between humans and animal models. Our observations provide insights into the possible mechanisms of cold-induced benefits on metabolic syndrome and neurodegenerative disorders in mice and elucidate targeting of specific lipid species for metabolic needs.

## Conclusion

We determined the cold-induced adaptive changes of lipids and genes in iWAT at the level of the transcriptome and lipidome. Our results revealed that cold adaption induces changes in the following: lipid composition, including glyphospholipids, glycerolipids, and sphingolipid in a subspecies-selective manner; the length of fatty-acyl chains associated with TAGs; and a markedly increased expression of genes involved in thermogenesis, fatty acid elongation, and fatty acid metabolism. Interestingly, we also found that some specific lipid species were negatively correlated to metabolic diseases, including obesity and T2D, suggesting that these lipid species may have potential roles in treating metabolic diseases. Future studies will be needed to determine the functional consequences or the exact roles of the specific remodeling of lipid species, as well as the increased expression of genes in iWAT.

## Methods

### Animals

All of the procedures involving mice were approved by the Zhejiang University Animal Care and Use Committee. Male C57BL6/J mice were single-housed under standard laboratory conditions, including a 12-h light/dark cycle, with free access to diet and water. Five mice were housed at either RT or at 4 °C for 3 days and were subsequently killed by cervical dislocation. iWAT and BAT were dissected, frozen immediately in liquid nitrogen, and stored at − 80 °C.

### Hematoxylin-eosin (H&E) staining

Brown and white adipose tissues from RT- and cold-treated mice were fixed in 10% formalin for 24 h at room temperature. Then, the tissues were embedded into paraffin, blocked, and cut at 5–10 μm for H&E staining. The sections were deparaffinized, rehydrated, and stained with hematoxylin for 15 min. Sections were then rinsed in running tap water and stained with eosin for 3–5 min, dehydrated, mounted, and captured.

### Total RNA extraction and quantitative real-time PCR

Total RNA extraction and real-time PCR were performed as previously described [108]. Briefly, total RNA was extracted from adipose tissues using Trizol Reagent (Thermo Fisher Scientific), and the purity and concentration of total RNA were measured. Two micrograms of total RNA was reverse-transcribed using random primers and MMLV reverse transcriptase (Thermo Fisher Scientific). Real-time PCR was carried out with an Applied Biosystems StepOnePlus™ Real-Time PCR System using SYBR Green Master Mix (Roche) and gene-specific primers (Additional file 15: Table S8). The 2^-ΔΔCT^ method was used to analyze the relative changes in gene expression normalized against 18S ribosomal RNA as an internal control.

### Protein extraction and western blotting

Total protein extraction and Western blotting were conducted as previously described [108]. Briefly, total protein was isolated from adipose tissues using RIPA buffer (50 mM Tris-HCl [pH 8.0], 150 mM NaCl, 1% NP-40, 0.5% sodium deoxycholate, and 0.1% SDS), and protein concentrations were determined using Pierce BCA Protein Assay Reagent (Thermo Fisher Scientific). Proteins were separated by SDS-PAGE, transferred to a polyvinylidene fluoride membrane (Millipore Corporation), and blocked in 5% fat-free milk for 1 h at RT, and they were then incubated with primary antibodies in 5% milk overnight at 4 °C. The UCP1 (Cat #: ab23841, Lot: GR3188478-15, RRID: AB_2213764, Dilution rate: 1:2000) antibody was purchased from Abcam, and the GAPDH (Cat #: EM1101, Lot: HG0718, RRID: AB_2811078, Dilution rate: 1:5000) antibody was purchased from HuaAn Biotechnology. Secondary antibodies (anti-rabbit IgG (Prod #: 31460, Lot #: RB230194, RRID: AB_228341, Invitrogen, Thermo Fisher Scientific) or anti-mouse IgG (Prod #: 31430, Lot #: RJ240410, RRID: AB_2040944, Invitrogen, Thermo Fisher Scientific) were diluted 5000-fold. Immunodetection was performed using an enhanced chemiluminescence western blotting substrate (Google Biotechnology) and detected with a ChemiScope3500 mini System.

### Lipid sample preparation and lipidomic assay

Lipid extraction and mass spectrometry-based lipid detection were performed by Applied Protein Technology. Take a separate sample in each group and mix them equally together to create a pooled QC sample. QC samples were inserted into the analysis queue to evaluate the system stability and data reliability during the whole experimental process. LC-MS/MS analysis was performed on a Q Exactive plus mass spectrometer (Thermo Scientific) coupled to a UHPLC Nexera LC-30A (SHIMADZU). Full-scan spectra were collected in mass-to-charge ratio (*m*/*z*) ranges of 200–1800 and 250–1800 for positive and negative ion modes, respectively. The mass-to-charge ratio of lipid molecules to lipid fragments was collected by the following method: after each full scan, 10 fragment patterns (MS2 scan, HCD) were collected. Lipid identification (secondary identification), peak extraction, peak alignment, and quantification were assessed with LipidSearch software version 4.1 (Thermo Scientific™). In the extracted ion features, only the variables having more than 50% of the nonzero measurement values in at least one group were kept. A complete list of the lipidomic data is provided in Additional file 2: Table S1.

### Unsupervised multivariate data analyses

For the multivariate statistical analysis, the SIMCA-P 14.1 software (Umeta, Umea, Sweden) was used. After the Pareto scaling, principal component analysis (PCA) and partial least-squares-discriminant analysis (PLS-DA) were performed. The leave-one-out cross-validation and response permutation testing were used to evaluate the robustness of the model. The significant different metabolites were determined based on the combination of a statistically significant threshold of variable influence on projection (VIP) values obtained from the PLS-DA model and two-tailed Student’s *t* test (*P* value) on the raw data, and the metabolites with VIP values larger than 1.0 and *P* values less than 0.1 were considered as significant.

Univariate analysis included the Student’s *t* test and variable fold change analysis. Hierarchical cluster analysis and correlation analysis were performed with R software (version 3.5.1). These values for all of the models are shown in Additional file 2: Table S1 and Additional file 4: Table S2. All the statistical evaluations using the PCA and OPLS methods described in this work were calculated from relative abundances.

### RNA-seq analysis

RNA extraction and RNA-seq analysis were performed by Sangon Biotech. Next, iWAT was extracted using the Total RNA Extractor (Trizol) kit (B511311, Sangon, China) according to the manufacturer’s protocol, and it was treated with RNase-free DNase I to remove genomic DNA contamination. A total amount of 2-μg RNA per sample was used as input material for the RNA sample preparations. Sequencing libraries were generated using VAHTSTM mRNA-seq V2 Library Prep Kit for Illumina®, following the manufacturer’s recommendations and index codes were added to attribute sequences to each sample. The libraries were then quantified and pooled. Paired-end sequencing of the library was performed on the HiSeq XTen sequencers (Illumina, San Diego, CA). FastQC (version 0.11.2) was used for evaluating the quality of sequenced data. Raw reads were filtered by Trimmomatic (version 0.36). Clean reads were mapped to the reference genome by HISAT2 (version 2.0) with default parameters. RSeQC (version 2.6.1) was used to run statistics on the alignment results. The homogeneity distribution and the genomic structure were checked by Qualimap (version 2.2.1). BEDTools (version 2.26.0) was used for statistical analysis of the gene coverage ratio. Gene expression values of the transcripts were computed by StringTie (version 1.3.3b). The TPM eliminated the influence of gene lengths and sequencing discrepancies to enable direct comparison of gene expression between samples. DESeq2 (version 1.12.4) was used to determine differentially expressed genes (DEGs) between two samples. Genes were considered as significantly differentially expressed if *q* value < 0.001 and |foldchange| > 1.5. A complete list of the transcriptome data is provided in Additional file 5: Table S3.

### Pathway enrichment assay

Functional enrichment analyses, including GO and KEGG, was performed to identify which DEGs were significantly enriched in GO terms or metabolic pathways. GO is an international standard classification system for gene function. DEGs are mapped to the GO terms (biological functions) in the database. The number of genes in every term is calculated, and a hypergeometric test is performed to identify significantly enriched GO terms in the gene list out of the background of the reference gene list. The KEGG database is a public database of pathway data. KEGG pathway analysis identifies significantly enriched metabolic pathways or signal transduction pathways enriched in DEGs compared to those of a reference gene background, using the hypergeometric test. GO terms and KEGG pathways with false discovery rates *P* < 0.05 were considered as significantly altered. The list of GO terms and KEGG pathway data are provided in Additional file 7: Table S4 and Additional file 8: Table S5.

### Correlational assay

All the correlations were calculated in R using method “spearman” or “pearson” for the Spearman’s or Pearson’s correlation coefficient, respectively. Pearson’s correlation is a measure of a linear correlation in the data, while Spearman’s correlation coefficient is based on the ranked values rather than the values themselves.

### Data analysis

All the statistical evaluations of lipidomic data described in this work were calculated from relative abundances. Experimental data are presented as the mean ± SEM. Comparisons were made by unpaired two-tailed Student’s *t* tests or one-way ANOVAs, as appropriate. Differences among groups were considered statistically significant at *P* < 0.05.

## Additional files

Additional file 1: **Figure S1.** Cold exposure for 3 days decreases the mass of adipose tissue and increases the expression of browning related genes. (a-c) Cold exposure for 3 days decreases the body weight gain (a, *n* = 8) and the mass of BAT, EWAT and iWAT (b, n = 8), but increases food intake (c, *n* = 4–5). (d) H&E staining of BAT and iWAT sections from control and cold-treated mice. Scale bars, 100 mm. (e) Western blots showing Ucp1 protein levels in BAT and IWAT. (f) mRNA of BAT- selective and adipocyte metabolism related genes in iWAT from control and cold-treated mice (*n* = 5). Error bars represent s.e.m. * *P* < 0.05, ** *P* < 0.01, *** *P* < 0.001, two-tailed Student’s t-test. (JPG 336 kb)

Additional file 2: Complete list of the lipidomic data (XLSX 707 kb)

Additional file 3: **Figure S2.** Multivariate data analysis for LC-MS lipidomics in control and cold-treated iWAT. (a) Quantified lipid classes and their abbreviations used throughout the paper. (b) Unsupervised principal component analysis (PCA) scores plot. Blue and green symbols represent RT and COLD iWAT samples, respectively. (c) Supervised OPLS-DA. Blue and green symbols represent RT and COLD samples, respectively. (d) Validation of the OPLS-DA model. (e) Heatmap of the significantly altered lipids (*P*-value < 0.05 and VIP > 1) in iWAT from control and cold-treated mice (n = 8). (JPG 2120 kb)

Additional file 4: Values for all of the models (XLSX 9 kb)

Additional file 5: Complete list of the transcriptome data (XLSX 10772 kb)

Additional file 6: **Figure S3.** Cold exposure induces BAT selective and lipid metabolism related genes. We compared the TPM expression values between the two cases to estimate relative gene expression abundance. (a) RNA-seq analysis shows the gene expression fold change of BAT-selective, pan-adipocyte and WAT- selective genes in IWAT from control (n = 5) and cold-treated (n = 4) mice. (b, c, d) RNA-seq analysis shows the gene expression fold change of glycerophospholipid metabolism, glycerolipid metabolism and fatty acid elongation related genes in IWAT from control (n = 5) and cold-treated (n = 4) mice. Error bars represent s.e.m. * *P* < 0.05, ** *P* < 0.01, *** *P* < 0.001, two-tailed Student’s t-test. (JPG 1403 kb)

Additional file 7: List of GO terms (XLS 3478 kb)

Additional file 8: Functional enrichment analyses using the Kyoto Encyclopedia of Genes and Genomes (KEGG) pathways (XLSX 11 kb)

Additional file 9: **Figure S4.** Cold exposure induces mRNA level of lipid metabolism related genes. (a-e) mRNA of glycerophospholipid metabolism, glycerolipid, fatty acid elongation, β-oxidation, sphingolipid metabolism related genes in iWAT from control and cold-treated mice (*n* = 6). Error bars represent s.e.m. * *P* < 0.05, ** *P* < 0.01, *** *P* < 0.001, two-tailed Student’s t-test. (JPG 1051 kb)

Additional file 10: **Figure S5.** Cold exposure changes the length of fatty acyl chains associated with TAG. (a) Percentage of SFA, MUFA and PUFA in TAG acyl chain in iWAT from control and cold-treated mice (*n* = 8). (b) Total MUFA to total PUFA ratio in TAG acyl chain. Error bars represent s.e.m.* *P* < 0.05, ** *P* < 0.01, *** *P* < 0.001, two-tailed Student’s t-test. (c) Correlation matrix for TAG acyl chain in iWAT based on Pearson’s correlation coefficient. (JPG 1111 kb)

Additional file 11: Transcription profiling by array of adipose samples from LDLR−/− mice to study response to anti-diabetic drug and dietary lifestyle interventions (XLSX 4553 kb)

Additional file 12: Transcript-lipidomic correlation network (XLSX 2590 kb)

Additional file 13: **Figure S6.** Transcript–lipidomics correlation network for the significantly changed genes and lipids induced by cold exposure. (a)Transcript-lipidomics correlation network analysis for significantly changed (*p* < 0.01) genes (green) and lipids (red) located based on Pearson’s correlation coefficient using Cytoscape. Red lines represent positive correlations and blue lines represent negative correlations. Thickness of each line has positive correlation with the absolute value of correlation. Size of each node has positive correlation with the value of Degree. (b, c) The top 20 genes (b) and top 20 lipids (c) with the greatest numbers amounts of correlated nodes. (JPG 4004 kb)

Additional file 14: **Figure S7.** Comparing of the lipidic programs in iWAT from cold-treated models and the plasma from insulin-resistant and obese/overweight patients. (a) The intensity of the lipid species that were reported to be significantly changed in insulin resistance patients. Green columns represent higher lipid species in insulin resistance patient. Yellow columns represent higher lipid species in insulin resistance patient. (b) The intensity of the lipid species that were reported to be markers for T2D. Green columns represent lipid species that might decrease risk of T2D. Green columns represent lipid species that might increase risk of T2D. (c) The intensity of the lipid species that were reported to be significantly changed during OGTT. Green columns represent lipid species that were increased during OGTT. Yellow columns represent lipid species that were decreased during OGTT. (d) The intensity of the lipid species that were reported to be markers for DM. Green columns represent lipid species that might decrease risk of DM. Green columns represent lipid species that might increase risk of DM. (e) The intensity of the lipid species that were reported to be significantly changed in overweight/obesity. Green columns represent decreased lipid species in overweight/obesity. Yellow columns represent increased lipid species in overweight/obesity. The transparency of each bar is proportional to the significance values, which are displayed as -log10 (*P*-value). (JPG 1667 kb)

Additional file 15: Gene-specific primers (XLSX 11 kb)

Additional file 16: Individual data values (XLSX 37 kb)

## Data Availability

All data generated or analyzed during this study are included in this published article and its supplementary information files. Additional datasets: Transcriptomics data from previous studies (GSE39549, Kwon et al. [109] and GSE57659, Radonjic et al. [110]) were obtained from GEO and analyzed. See Additional file 11: Table S6 for more details. The individual data values are provided in Additional file 16: Table S9.

## References

[1] Oelkrug R, Polymeropoulos ET, Jastroch M (2015). Brown adipose tissue: physiological function and evolutionary significance. J Comp Physiol B.

[2] Fabbiano S, Suarez-Zamorano N, Rigo D, Veyrat-Durebex C, Stevanovic Dokic A, Colin DJ (2016). Caloric restriction leads to browning of white adipose tissue through type 2 immune signaling. Cell Metab.

[3] Fisher FM, Kleiner S, Douris N, Fox EC, Mepani RJ, Verdeguer F (2012). FGF21 regulates PGC-1alpha and browning of white adipose tissues in adaptive thermogenesis. Genes Dev.

[4] Jun W, Paul C, Spiegelman BM (2013). Adaptive thermogenesis in adipocytes: is beige the new brown?. Genes Dev.

[5] Agabiti-Rosei C, De Ciuceis C, Rossini C, Porteri E, Rodella LF, Withers SB (2014). Anticontractile activity of perivascular fat in obese mice and the effect of long-term treatment with melatonin. J Hypertens.

[6] Patrick S, Conroe HM, Jennifer E, Shingo K, Andrea F, Jeff I (2011). Prdm16 determines the thermogenic program of subcutaneous white adipose tissue in mice. J Clin Invest.

[7] Shabalina IG, Petrovic N, de Jong JM, Kalinovich AV, Cannon B, Nedergaard J (2013). UCP1 in brite/beige adipose tissue mitochondria is functionally thermogenic. Cell Rep.

[8] Seale P, Bjork B, Yang W, Kajimura S, Chin S, Kuang S (2008). PRDM16 controls a brown fat/skeletal muscle switch. Nature..

[9] Natasa P, Walden TB, Shabalina IG, Timmons JA, Barbara C, Jan N (2010). Chronic peroxisome proliferator-activated receptor gamma (PPARgamma) activation of epididymally derived white adipocyte cultures reveals a population of thermogenically competent, UCP1-containing adipocytes molecularly distinct from classic brown adipocyte. J Biol Chem.

[10] Wu J, Bostrom P, Sparks LM, Ye L, Choi JH, Giang AH (2012). Beige adipocytes are a distinct type of thermogenic fat cell in mouse and human. Cell..

[11] Bi P, Shan T, Liu W, Yue F, Yang X, Liang XR (2014). Inhibition of Notch signaling promotes browning of white adipose tissue and ameliorates obesity. Nat Med.

[12] Crane JD, Palanivel R, Mottillo EP, Bujak AL, Wang H, Ford RJ (2015). Inhibiting peripheral serotonin synthesis reduces obesity and metabolic dysfunction by promoting brown adipose tissue thermogenesis. Nat Med.

[13] Sharon L, Jennifer H, Yuan X, Takahiro S, Ziquan C, Patrik A (2012). Cold-induced activation of brown adipose tissue and adipose angiogenesis in mice. Nat Protoc.

[14] van der Lans AA, Hoeks J, Brans B, Vijgen GH, Visser MG, Vosselman MJ (2013). Cold acclimation recruits human brown fat and increases nonshivering thermogenesis. J Clin Invest.

[15] Marcher AB, Loft A, Nielsen R, Vihervaara T, Madsen JG, Sysi-Aho M (2015). RNA-Seq and mass-spectrometry-based lipidomics reveal extensive changes of glycerolipid pathways in brown adipose tissue in response to cold. Cell Rep.

[16] Lu X, Solmonson A, Lodi A, Nowinski SM, Sentandreu E, Riley CL (2017). The early metabolomic response of adipose tissue during acute cold exposure in mice. Sci Rep.

[17] Lynes MD, Leiria LO, Lundh M, Bartelt A, Shamsi F, Huang TL (2017). The cold-induced lipokine 12,13-diHOME promotes fatty acid transport into brown adipose tissue. Nat Med.

[18] Simcox J, Geoghegan G, Maschek JA, Bensard CL, Pasquali M, Miao R (2017). Global analysis of plasma lipids identifies liver-derived acylcarnitines as a fuel source for brown fat thermogenesis. Cell Metab.

[19] Bai Z, Chai XR, Yoon MJ, Kim HJ, Lo KA, Zhang ZC (2017). Dynamic transcriptome changes during adipose tissue energy expenditure reveal critical roles for long noncoding RNA regulators. PLoS Biol.

[20] Lynes MD, Shamsi F, Sustarsic EG, Leiria LO, Wang CH, Su SC (2018). Cold-activated lipid dynamics in adipose tissue highlights a role for cardiolipin in thermogenic metabolism. Cell Rep.

[21] Sustarsic EG, Ma T, Lynes MD, Larsen M, Karavaeva I, Havelund JF (2018). Cardiolipin synthesis in brown and beige fat mitochondria is essential for systemic energy homeostasis. Cell Metab.

[22] Cannon B, Nedergaard J (2004). Brown adipose tissue: function and physiological significance. Physiol Rev.

[23] Stephenson DJ, Hoeferlin LA, Chalfant CE (2017). Lipidomics in translational research and the clinical significance of lipid-based biomarkers. Transl Res.

[24] Hansen M, Flatt T, Aguilaniu H (2013). Reproduction, fat metabolism, and life span: what is the connection?. Cell Metab.

[25] Li G, Xie C, Lu S, Nichols RG, Tian Y, Li L (2017). Intermittent fasting promotes white adipose browning and decreases obesity by shaping the gut microbiota. Cell Metab.

[26] Fischer C, Seki T, Lim S, Nakamura M, Andersson P, Yang Y (2017). A miR-327-FGF10-FGFR2-mediated autocrine signaling mechanism controls white fat browning. Nat Commun.

[27] Nedergaard J, Bengtsson T, Cannon B (2007). Unexpected evidence for active brown adipose tissue in adult humans. Am J Physiol Endocrinol Metab.

[28] Rossato M (2016). Aging and brown adipose tissue activity decline in human: does the brain extinguish the fire?. Aging Clin Exp Res.

[29] Enerback S (2010). Human brown adipose tissue. Cell Metab.

[30] Loeb J, Northrop JH (1916). Is there a temperature coefficient for the duration of life?. P Natl Acad Sci USA.

[31] Conti B, Sanchez-Alavez M, Winsky-Sommerer R, Morale MC, Lucero J, Brownell S (2006). Transgenic mice with a reduced core body temperature have an increased life span. Science..

[32] Speakman JR, Keijer J (2012). Not so hot: optimal housing temperatures for mice to mimic the thermal environment of humans. Mol Metab.

[33] Fischer AW, Cannon B, Nedergaard J (2018). Optimal housing temperatures for mice to mimic the thermal environment of humans: an experimental study. Mol Metab..

[34] Plaisier CL, Bennett BJ, He A, Guan B, Lusis AJ, Reue K (2012). Zbtb16 has a role in brown adipocyte bioenergetics. Nutr Diabetes.

[35] Shore AM, Karamitri A, Kemp P, Speakman JR, Graham NS, Lomax MA (2013). Cold-induced changes in gene expression in brown adipose tissue, white adipose tissue and liver. PLoS One.

[36] Rosell M, Kaforou M, Frontini A, Okolo A, Chan YW, Nikolopoulou E (2014). Brown and white adipose tissues: intrinsic differences in gene expression and response to cold exposure in mice. Am J Physiol Endocrinol Metab.

[37] Hao Q, Yadav R, Basse AL, Petersen S, Sonne SB, Rasmussen S (2015). Transcriptome profiling of brown adipose tissue during cold exposure reveals extensive regulation of glucose metabolism. Am J Physiol Endocrinol Metab.

[38] Xu Z, Liu J, You W, Wang Y, Shan T (2019). Cold exposure induces nuclear translocation of CRTC3 in brown adipose tissue. J Cell Biochem.

[39] Kanehisa M, Goto S (2000). KEGG: Kyoto Encyclopedia of Genes and Genomes. Nucleic Acids Res.

[40] Kanehisa M, Goto S, Sato Y, Kawashima M, Furumichi M, Tanabe M (2014). Data, information, knowledge and principle: back to metabolism in KEGG. Nucleic Acids Res.

[41] Gonzalez-Covarrubias V, Beekman M, Uh HW, Dane A, Troost J, Paliukhovich I (2013). Lipidomics of familial longevity. Aging Cell.

[42] Shmookler Reis RJ, Xu L, Lee H, Chae M, Thaden JJ, Bharill P (2011). Modulation of lipid biosynthesis contributes to stress resistance and longevity of C. elegans mutants. Aging..

[43] Stegemann C, Pechlaner R, Willeit P, Langley SR, Mangino M, Mayr U (2014). Lipidomics profiling and risk of cardiovascular disease in the prospective population-based Bruneck study. Circulation..

[44] Rhee EP, Cheng S, Larson MG, Walford GA, Lewis GD, McCabe E (2011). Lipid profiling identifies a triacylglycerol signature of insulin resistance and improves diabetes prediction in humans. J Clin Invest.

[45] Kihara A (2012). Very long-chain fatty acids: elongation, physiology and related disorders. J Biochem.

[46] Ohno Y, Suto S, Yamanaka M, Mizutani Y, Mitsutake S, Igarashi Y (2010). ELOVL1 production of C24 acyl-CoAs is linked to C24 sphingolipid synthesis. Proc Natl Acad Sci U S A.

[47] Naganuma T, Sato Y, Sassa T, Ohno Y, Kihara A (2011). Biochemical characterization of the very long-chain fatty acid elongase ELOVL7. FEBS Lett.

[48] Guillou H, Zadravec D, Martin PG, Jacobsson A (2010). The key roles of elongases and desaturases in mammalian fatty acid metabolism: insights from transgenic mice. Prog Lipid Res.

[49] Hishikawa D, Hashidate T, Shimizu T, Shindou H (2014). Diversity and function of membrane glycerophospholipids generated by the remodeling pathway in mammalian cells. J Lipid Res.

[50] Hoene M, Li J, Haring HU, Weigert C, Xu G, Lehmann R (2014). The lipid profile of brown adipose tissue is sex-specific in mice. Biochim Biophys Acta.

[51] Schunck WH, Konkel A, Fischer R, Weylandt KH (2018). Therapeutic potential of omega-3 fatty acid-derived epoxyeicosanoids in cardiovascular and inflammatory diseases. Pharmacol Ther.

[52] Meikle PJ, Summers SA (2017). Sphingolipids and phospholipids in insulin resistance and related metabolic disorders. Nat Rev Endocrinol.

[53] Alexaki A, Clarke BA, Gavrilova O, Ma YY, Zhu HL, Ma XR (2017). De novo sphingolipid biosynthesis is required for adipocyte survival and metabolic homeostasis. J Biol Chem.

[54] Holland WL, Brozinick JT, Wang LP, Hawkins ED, Sargent KM, Liu Y (2007). Inhibition of ceramide synthesis ameliorates glucocorticoid-, saturated-fat-, and obesity-induced insulin resistance. Cell Metab.

[55] Turpin SM, Nicholls HT, Willmes DM, Mourier A, Brodesser S, Wunderlich CM (2014). Obesity-induced CerS6-dependent C16:0 ceramide production promotes weight gain and glucose intolerance. Cell Metab.

[56] Cheng LI, Jin-Zhi HE, Zhou XD, Xin XU (2017). Berberine regulates type 2 diabetes mellitus related with insulin resistance. China J Chin Mater Med.

[57] Wilson DF (2017). Oxidative phosphorylation: unique regulatory mechanism and role in metabolic homeostasis. J Appl Physiol (1985).

[58] Jia Y, Hong J, Li H, Hu Y, Jia L, Cai D (2017). Butyrate stimulates adipose lipolysis and mitochondrial oxidative phosphorylation through histone hyperacetylation-associated beta3-adrenergic receptor activation in high-fat diet-induced obese mice. Exp Physiol.

[59] Gray LR, Tompkins SC, Taylor EB (2014). Regulation of pyruvate metabolism and human disease. Cell Mol Life Sci.

[60] Kwon EY, Shin SK, Cho YY, Jung UJ, Kim E, Park T (2012). Time-course microarrays reveal early activation of the immune transcriptome and adipokine dysregulation leads to fibrosis in visceral adipose depots during diet-induced obesity. BMC Genomics.

[61] Radonjic M, Wielinga PY, Wopereis S, Kelder T, Goelela VS, Verschuren L (2013). Differential effects of drug interventions and dietary lifestyle in developing type 2 diabetes and complications: a systems biology analysis in LDLr−/− mice. PLoS One.

[62] van den Beukel JC, Boon MR, Steenbergen J, Rensen PC, Meijer OC, Themmen AP (2015). Cold exposure partially corrects disturbances in lipid metabolism in a male mouse model of glucocorticoid excess. Endocrinology..

[63] Kandimalla R, Thirumala V, Reddy PH (2017). Is Alzheimer’s disease a type 3 diabetes? A critical appraisal. Biochim Biophys Acta Mol basis Dis.

[64] Shinohara M, Sato N (2017). Bidirectional interactions between diabetes and Alzheimer’s disease. Neurochem Int.

[65] Leszek J, Trypka E, Tarasov VV, Md AG (2017). Type 3 diabetes mellitus: a novel implication of Alzheimer disease. Curr Top Med Chem.

[66] de la Monte SM (2017). Insulin resistance and neurodegeneration: progress towards the development of new therapeutics for Alzheimer’s disease. Drugs..

[67] Pardeshi R, Bolshette N, Gadhave K, Ahire A, Ahmed S, Cassano T (2017). Insulin signaling: an opportunistic target to minify the risk of Alzheimer’s disease. Psychoneuroendocrinology.

[68] Claxton A, Baker LD, Hanson A, Trittschuh EH, Cholerton B, Morgan A (2015). Long-acting intranasal insulin detemir improves cognition for adults with mild cognitive impairment or early-stage Alzheimer’s disease dementia. J Alzheimers Dis.

[69] Sato T, Morita A, Mori N, Miura S (2014). The role of glycerol-3-phosphate dehydrogenase 1 in the progression of fatty liver after acute ethanol administration in mice. Biochem Biophys Res Commun.

[70] Coleman RA, Lee DP (2004). Enzymes of triacylglycerol synthesis and their regulation. Prog Lipid Res.

[71] Moolenaar WH, van Meeteren LA, Giepmans BN (2004). The ins and outs of lysophosphatidic acid signaling. Bioessays..

[72] Vergeer M, Cohn DM, Boekholdt SM, Sandhu MS, Prins HM, Ricketts SL (2010). Lack of association between common genetic variation in endothelial lipase (LIPG) and the risk for CAD and DVT. Atherosclerosis..

[73] Osman C, Voelker DR, Langer T (2011). Making heads or tails of phospholipids in mitochondria. J Cell Biol.

[74] Daum G (1985). Lipids of mitochondria. Biochim Biophys Acta.

[75] van Meer G, Voelker DR, Feigenson GW (2008). Membrane lipids: where they are and how they behave. Nat Rev Mol Cell Bio.

[76] Exil VJ, Gardner CD, Rottman JN, Sims H, Bartelds B, Khuchua Z (2006). Abnormal mitochondrial bioenergetics and heart rate dysfunction in mice lacking very-long-chain acyl-CoA dehydrogenase. Am J Physiol Heart Circ Physiol.

[77] Tucci S, Behringer S, Spiekerkoetter U (2015). De novo fatty acid biosynthesis and elongation in very long-chain acyl-CoA dehydrogenase-deficient mice supplemented with odd or even medium-chain fatty acids. FEBS J.

[78] Hodson L, Eyles HC, McLachlan KJ, Bell ML, Green TJ, Skeaff CM (2014). Plasma and erythrocyte fatty acids reflect intakes of saturated and n-6 PUFA within a similar time frame. J Nutr.

[79] Hodge AM, English DR, O'Dea K, Sinclair AJ, Makrides M, Gibson RA (2007). Plasma phospholipid and dietary fatty acids as predictors of type 2 diabetes: interpreting the role of linoleic acid. Am J Clin Nutr.

[80] Patel PS, Sharp SJ, Jansen E, Luben RN, Khaw KT, Wareham NJ (2010). Fatty acids measured in plasma and erythrocyte-membrane phospholipids and derived by food-frequency questionnaire and the risk of new-onset type 2 diabetes: a pilot study in the European Prospective Investigation into Cancer and Nutrition (EPIC)-Norfolk cohort. Am J Clin Nutr.

[81] Mozaffarian D, Cao H, King IB, Lemaitre RN, Song X, Siscovick DS (2010). Trans-palmitoleic acid, metabolic risk factors, and new-onset diabetes in U.S. adults: a cohort study. Ann Intern Med.

[82] Mozaffarian D, de Oliveira Otto MC, Lemaitre RN, Fretts AM, Hotamisligil G, Tsai MY (2013). *trans*-Palmitoleic acid, other dairy fat biomarkers, and incident diabetes: the Multi-Ethnic Study of Atherosclerosis (MESA). Am J Clin Nutr.

[83] Santaren ID, Watkins SM, Liese AD, Wagenknecht LE, Rewers MJ, Haffner SM (2014). Serum pentadecanoic acid (15:0), a short-term marker of dairy food intake, is inversely associated with incident type 2 diabetes and its underlying disorders. Am J Clin Nutr.

[84] Sun Q, Ma J, Campos H, Hu FB (2007). Plasma and erythrocyte biomarkers of dairy fat intake and risk of ischemic heart disease. Am J Clin Nutr.

[85] Warensjo E, Jansson JH, Berglund L, Boman K, Ahren B, Weinehall L (2004). Estimated intake of milk fat is negatively associated with cardiovascular risk factors and does not increase the risk of a first acute myocardial infarction. A prospective case-control study. Br J Nutr.

[86] Warensjo E, Jansson JH, Cederholm T, Boman K, Eliasson M, Hallmans G (2010). Biomarkers of milk fat and the risk of myocardial infarction in men and women: a prospective, matched case-control study. Am J Clin Nutr.

[87] Roberts LD, Virtue S, Vidal-Puig A, Nicholls AW, Griffin JL (2009). Metabolic phenotyping of a model of adipocyte differentiation. Physiol Genomics.

[88] Gotoh N, Moroda K, Watanabe H, Yoshinaga K, Tanaka M, Mizobe H (2008). Metabolism of odd-numbered fatty acids and even-numbered fatty acids in mouse. J Oleo Sci.

[89] Pfeuffer M, Jaudszus A (2016). Pentadecanoic and heptadecanoic acids: multifaceted odd-chain fatty acids. Adv Nutr.

[90] Kulkarni H, Mamtani M, Wong G, Weir JM, Barlow CK, Dyer TD (2017). Genetic correlation of the plasma lipidome with type 2 diabetes, prediabetes and insulin resistance in Mexican American families. BMC Genet.

[91] Khairallah RJ, Sparagna GC, Khanna N, O'Shea KM, Hecker PA, Kristian T (2010). Dietary supplementation with docosahexaenoic acid, but not eicosapentaenoic acid, dramatically alters cardiac mitochondrial phospholipid fatty acid composition and prevents permeability transition. Biochim Biophys Acta.

[92] Yurko-Mauro K (2010). Cognitive and cardiovascular benefits of docosahexaenoic acid in aging and cognitive decline. Curr Alzheimer Res.

[93] Kitson AP, Metherel AH, Chen CT, Domenichiello AF, Trepanier MO, Berger A (2016). Effect of dietary docosahexaenoic acid (DHA) in phospholipids or triglycerides on brain DHA uptake and accretion. J Nutr Biochem.

[94] Blachnio-Zabielska AU, Baranowski M, Hirnle T, Zabielski P, Lewczuk A, Dmitruk I (2012). Increased bioactive lipids content in human subcutaneous and epicardial fat tissue correlates with insulin resistance. Lipids..

[95] Hinterwirth H, Stegemann C, Mayr M (2014). Lipidomics: quest for molecular lipid biomarkers in cardiovascular disease. Circ Cardiovasc Genet.

[96] Kolak M, Gertow J, Westerbacka J, Summers SA, Liska J, Franco-Cereceda A (2012). Expression of ceramide-metabolising enzymes in subcutaneous and intra-abdominal human adipose tissue. Lipids Health Dis.

[97] Chaurasia B, Kaddai VA, Lancaster GI, Henstridge DC, Sriram S, Galam DL (2016). Adipocyte ceramides regulate subcutaneous adipose browning, inflammation, and metabolism. Cell Metab.

[98] Mielke MM, Haughey NJ, Han D, An Y, Bandaru VVR, Lyketsos CG (2017). The association between plasma ceramides and sphingomyelins and risk of Alzheimer’s disease differs by sex and APOE in the Baltimore Longitudinal Study of Aging. J Alzheimers Dis.

[99] Fahumiya S, Hester KD, Guang Y, Hannun YA, Jacek B (2006). Altered adipose and plasma sphingolipid metabolism in obesity: a potential mechanism for cardiovascular and metabolic risk. Diabetes.

[100] Memon RA, Holleran WM, Moser AH, Seki T, Uchida Y, Fuller J (1998). Endotoxin and cytokines increase hepatic sphingolipid biosynthesis and produce lipoproteins enriched in ceramides and sphingomyelin. Arterioscler Thromb Vasc Biol.

[101] Wohlgemuth SE, Calvani R, Marzetti E (2014). The interplay between autophagy and mitochondrial dysfunction in oxidative stress-induced cardiac aging and pathology. J Mol Cell Cardiol.

[102] Marzetti E, Csiszar A, Dutta D, Balagopal G, Calvani R, Leeuwenburgh C (2013). Role of mitochondrial dysfunction and altered autophagy in cardiovascular aging and disease: from mechanisms to therapeutics. Am J Physiol Heart Circ Physiol.

[103] Tournissac M, Vandal M, Francois A, Planel E, Calon F (2017). Old age potentiates cold-induced tau phosphorylation: linking thermoregulatory deficit with Alzheimer’s disease. Neurobiol Aging.

[104] Tournissac M, Bourassa P, Martinez-Cano RD, Vu TM, Hebert SS, Planel E (2019). Repeated cold exposures protect a mouse model of Alzheimer’s disease against cold-induced tau phosphorylation. Mol Metab.

[105] Letra L, Santana I (2017). The influence of adipose tissue on brain development, cognition, and risk of neurodegenerative disorders. Adv Neurobiol.

[106] Zhao X, Peter A, Fritsche J, Elcnerova M, Fritsche A, Haring HU (2009). Changes of the plasma metabolome during an oral glucose tolerance test: is there more than glucose to look at?. Am J Physiol Endocrinol Metab.

[107] Floegel A, Stefan N, Yu Z, Muhlenbruch K, Drogan D, Joost HG (2013). Identification of serum metabolites associated with risk of type 2 diabetes using a targeted metabolomic approach. Diabetes..

[108] Shan T, Xiong Y, Zhang P, Li Z, Jiang Q, Bi P (2016). Lkb1 controls brown adipose tissue growth and thermogenesis by regulating the intracellular localization of CRTC3. Nat Commun.

[109] Kwon E, Choi M. Time-course microarrays reveal early activation of the immune transcriptome and adipokine dysregulation leads to fibrosis in visceral adipose depots during diet-induced obesity. GEO. 2014. https://www.ncbi.nlm.nih.gov/geo/query/acc.cgi?acc=GSE39549. Accessed 26 Feb 2019. 10.1186/1471-2164-13-450 PMC3447724 22947075

[110] Radonjic M WP, Kelder T, Goelela VS, Verschuren L, van Duyvenvoorde W, Stroeve JH, Cnubben N, Kooistra T, van Ommen B, Kleemann R.Transcription profiling by array of adipose samples from LDLR−/− mice to study response to anti-diabetic drug and dietary lifestyle interventions. GEO. 2014.https://www.ncbi.nlm.nih.gov/geo/query/acc.cgi?acc=GSE57659. Accessed 26 Feb 2019.

