# Supplementary figures and images for: Cold-induced lipid dynamics and transcriptional programs in white adipose tissue
Source: BMC Biol. 2019 Sep 17;17:74. doi: 10.1186/s12915-019-0693-x (PMC6749700; doi:10.1186/s12915-019-0693-x)

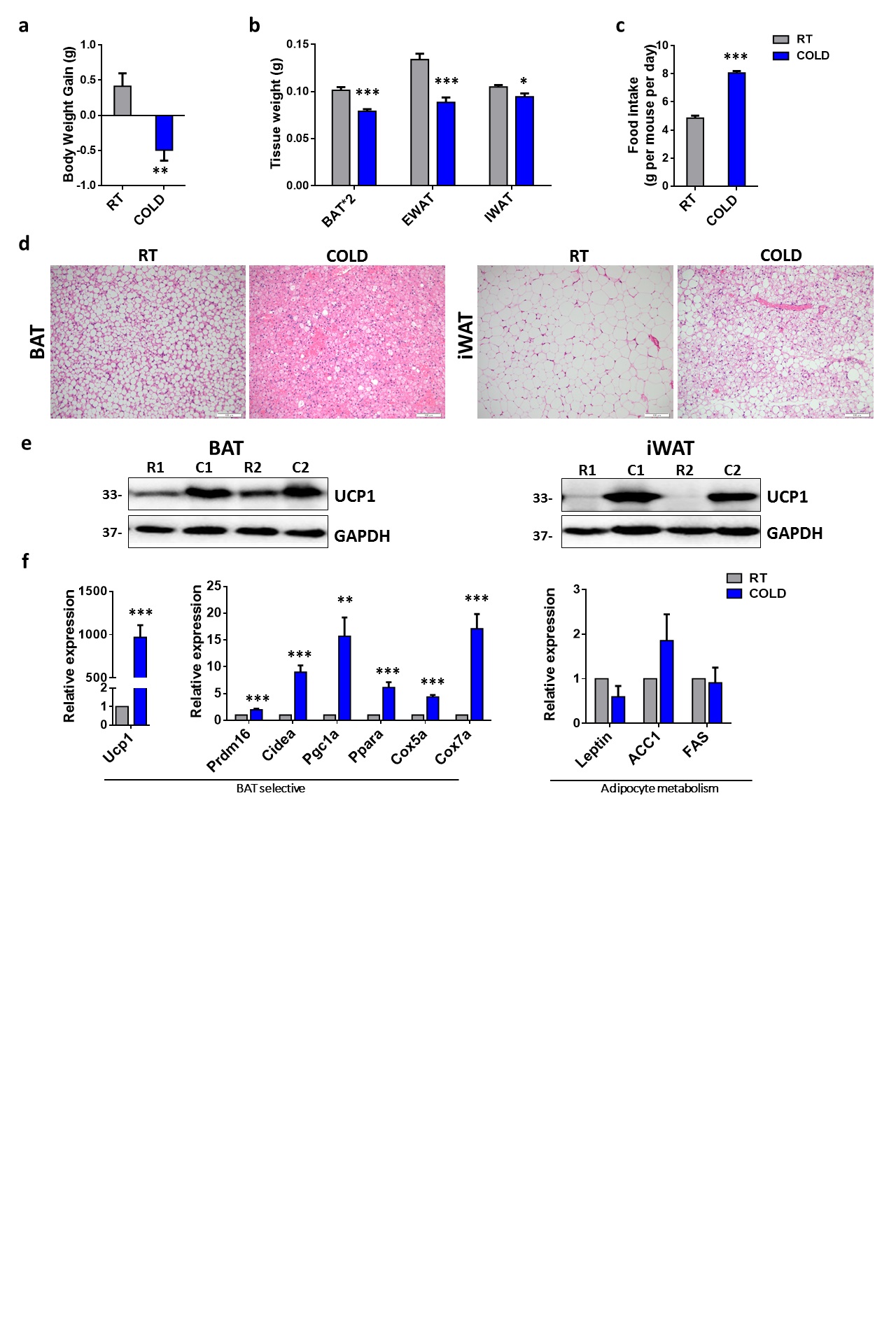

Supplement: Supplementary file 1 — Figure S1. Cold exposure for 3 days decreases the mass of adipose tissue and increases the expression of browning related genes. (a-c) Cold exposure for 3 days decreases the body weight gain (a, n = 8) and the mass of BAT, EWAT and iWAT (b, n = 8), but increases food intake (c, n = 4–5). (d) H&E staining of BAT and iWAT sections from control and cold-treated mice. Scale bars, 100 mm. (e) Western blots showing Ucp1 protein levels in BAT and IWAT. (f) mRNA of BAT- selective and adipocyte metabolism related genes in iWAT from control and cold-treated mice (n = 5). Error bars represent s.e.m. * P < 0.05, ** P < 0.01, *** P < 0.001, two-tailed Student’s t-test. (JPG 336 kb) [file 12915_2019_693_MOESM1_ESM.jpg]

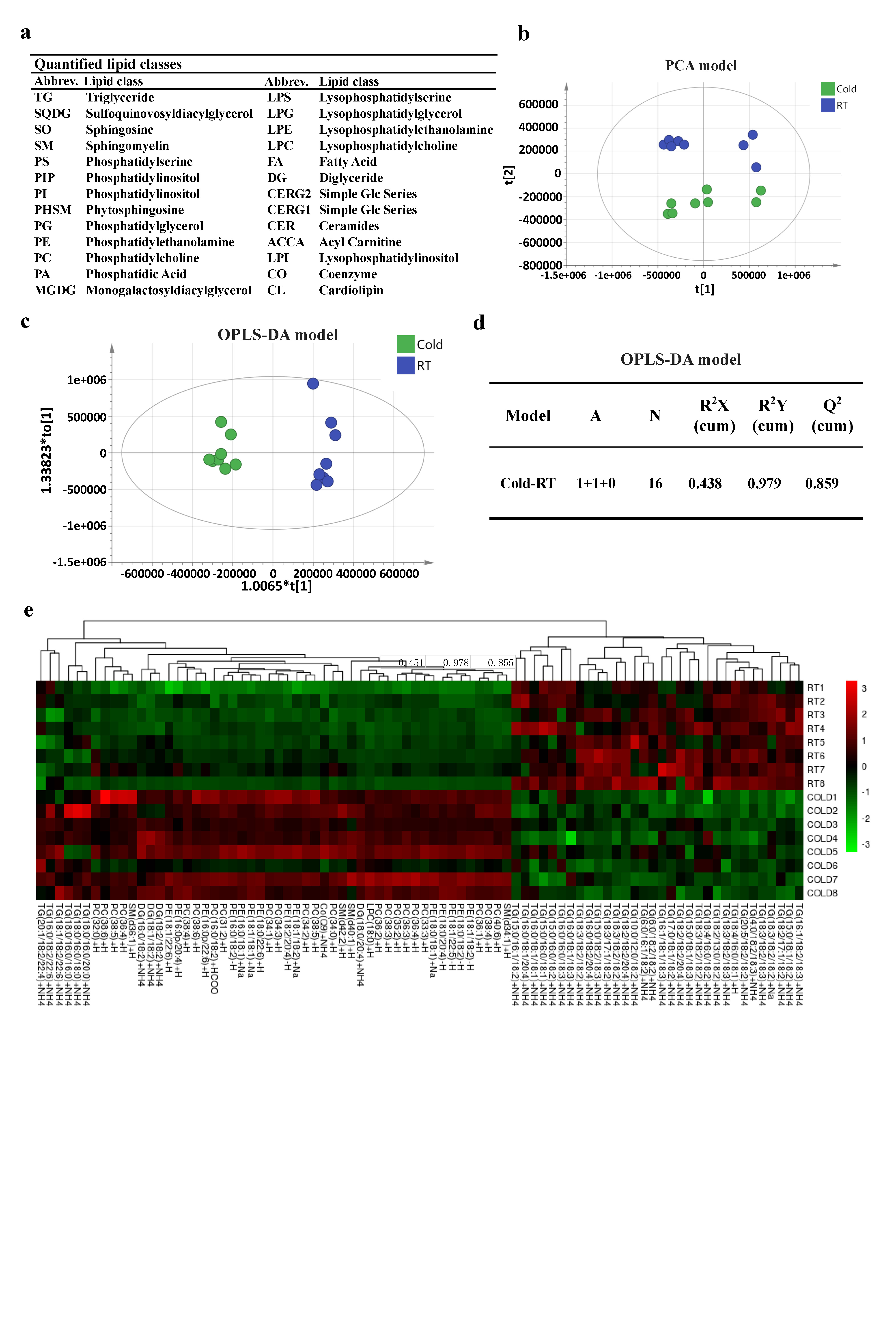

Supplement: Supplementary file 3 — Figure S2. Multivariate data analysis for LC-MS lipidomics in control and cold-treated iWAT. (a) Quantified lipid classes and their abbreviations used throughout the paper. (b) Unsupervised principal component analysis (PCA) scores plot. Blue and green symbols represent RT and COLD iWAT samples, respectively. (c) Supervised OPLS-DA. Blue and green symbols represent RT and COLD samples, respectively. (d) Validation of the OPLS-DA model. (e) Heatmap of the significantly altered lipids (P-value < 0.05 and VIP > 1) in iWAT from control and cold-treated mice (n = 8). (JPG 2120 kb) [file 12915_2019_693_MOESM3_ESM.jpg]

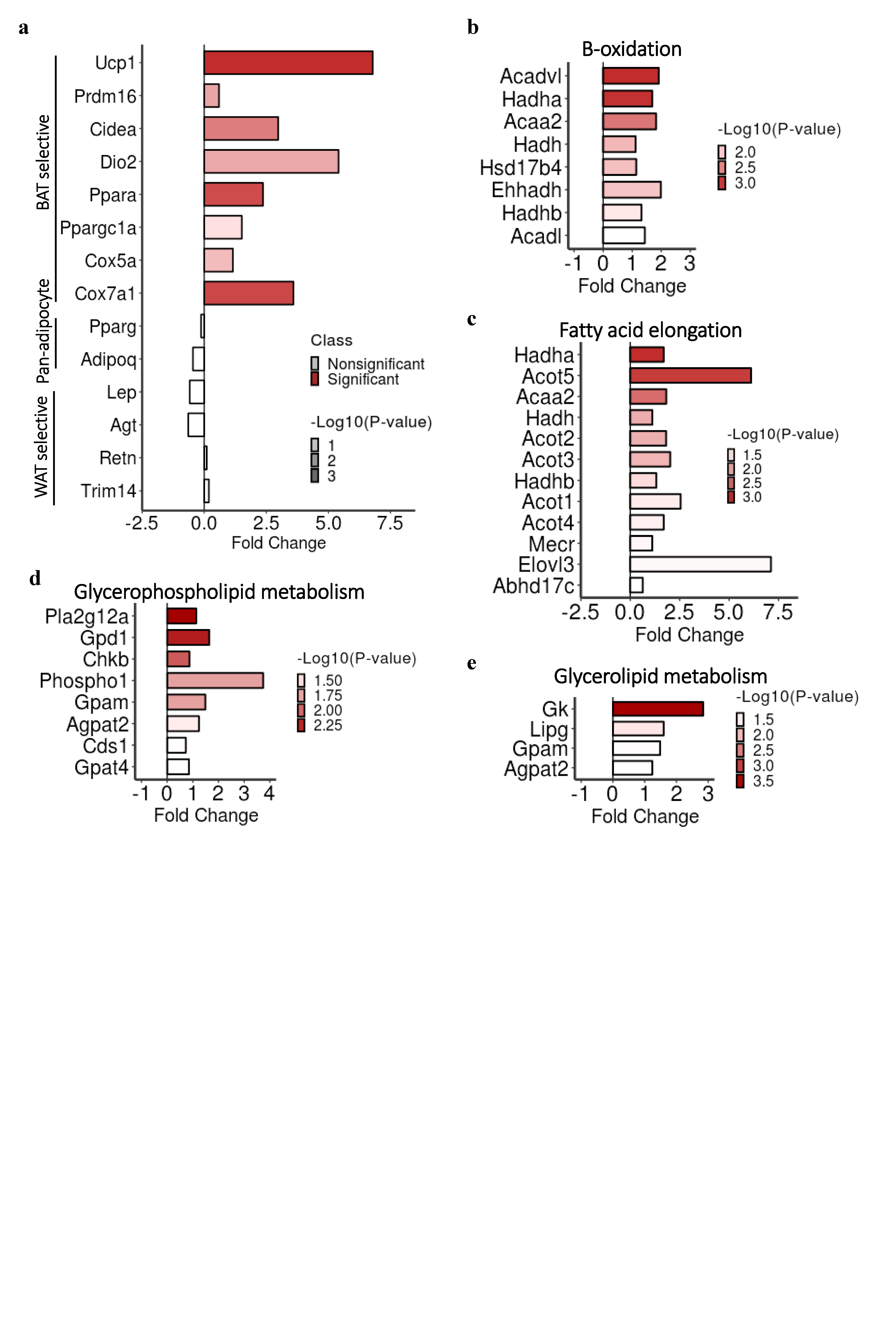

Supplement: Supplementary file 6 — Figure S3. Cold exposure induces BAT selective and lipid metabolism related genes. We compared the TPM expression values between the two cases to estimate relative gene expression abundance. (a) RNA-seq analysis shows the gene expression fold change of BAT-selective, pan-adipocyte and WAT- selective genes in IWAT from control (n = 5) and cold-treated (n = 4) mice. (b, c, d) RNA-seq analysis shows the gene expression fold change of glycerophospholipid metabolism, glycerolipid metabolism and fatty acid elongation related genes in IWAT from control (n = 5) and cold-treated (n = 4) mice. Error bars represent s.e.m. * P < 0.05, ** P < 0.01, *** P < 0.001, two-tailed Student’s t-test. (JPG 1403 kb) [file 12915_2019_693_MOESM6_ESM.jpg]

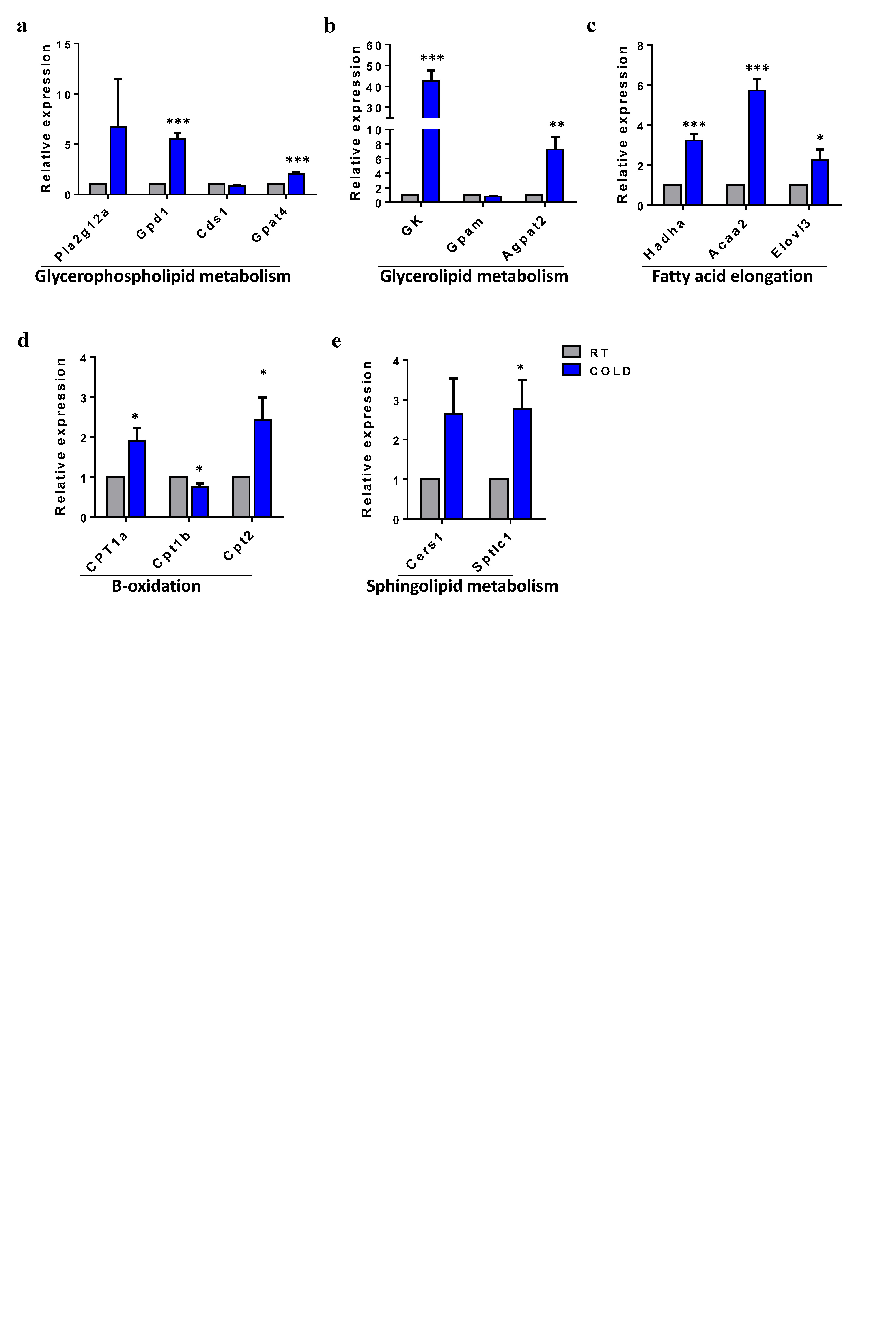

Supplement: Supplementary file 9 — Figure S4. Cold exposure induces mRNA level of lipid metabolism related genes. (a-e) mRNA of glycerophospholipid metabolism, glycerolipid, fatty acid elongation, β-oxidation, sphingolipid metabolism related genes in iWAT from control and cold-treated mice (n = 6). Error bars represent s.e.m. * P < 0.05, ** P < 0.01, *** P < 0.001, two-tailed Student’s t-test. (JPG 1051 kb) [file 12915_2019_693_MOESM9_ESM.jpg]

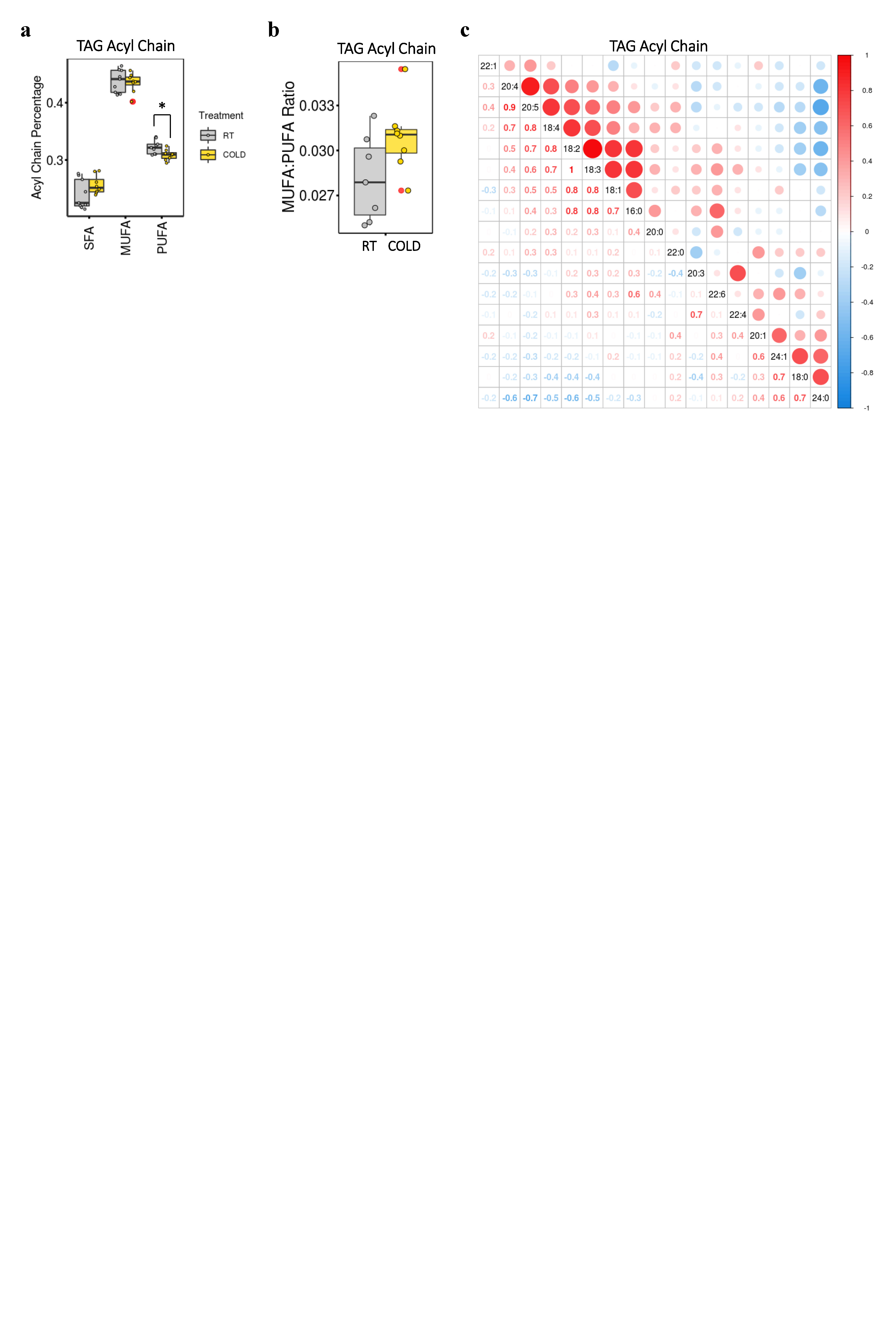

Supplement: Supplementary file 10 — Figure S5. Cold exposure changes the length of fatty acyl chains associated with TAG. (a) Percentage of SFA, MUFA and PUFA in TAG acyl chain in iWAT from control and cold-treated mice (n = 8). (b) Total MUFA to total PUFA ratio in TAG acyl chain. Error bars represent s.e.m.* P < 0.05, ** P < 0.01, *** P < 0.001, two-tailed Student’s t-test. (c) Correlation matrix for TAG acyl chain in iWAT based on Pearson’s correlation coefficient. (JPG 1111 kb) [file 12915_2019_693_MOESM10_ESM.jpg]

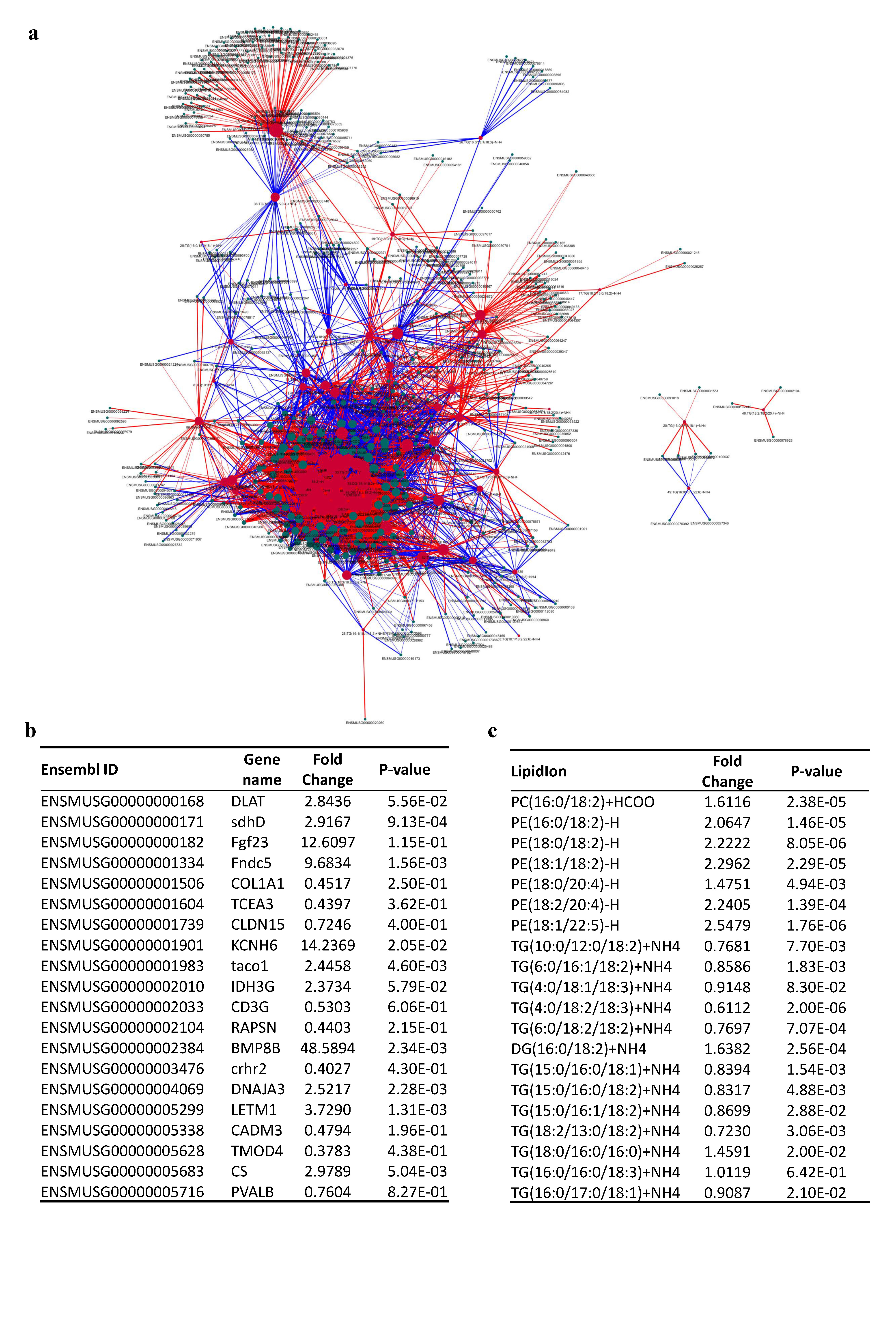

Supplement: Supplementary file 13 — Figure S6. Transcript–lipidomics correlation network for the significantly changed genes and lipids induced by cold exposure. (a)Transcript-lipidomics correlation network analysis for significantly changed (p < 0.01) genes (green) and lipids (red) located based on Pearson’s correlation coefficient using Cytoscape. Red lines represent positive correlations and blue lines represent negative correlations. Thickness of each line has positive correlation with the absolute value of correlation. Size of each node has positive correlation with the value of Degree. (b, c) The top 20 genes (b) and top 20 lipids (c) with the greatest numbers amounts of correlated nodes. (JPG 4004 kb) [file 12915_2019_693_MOESM13_ESM.jpg]

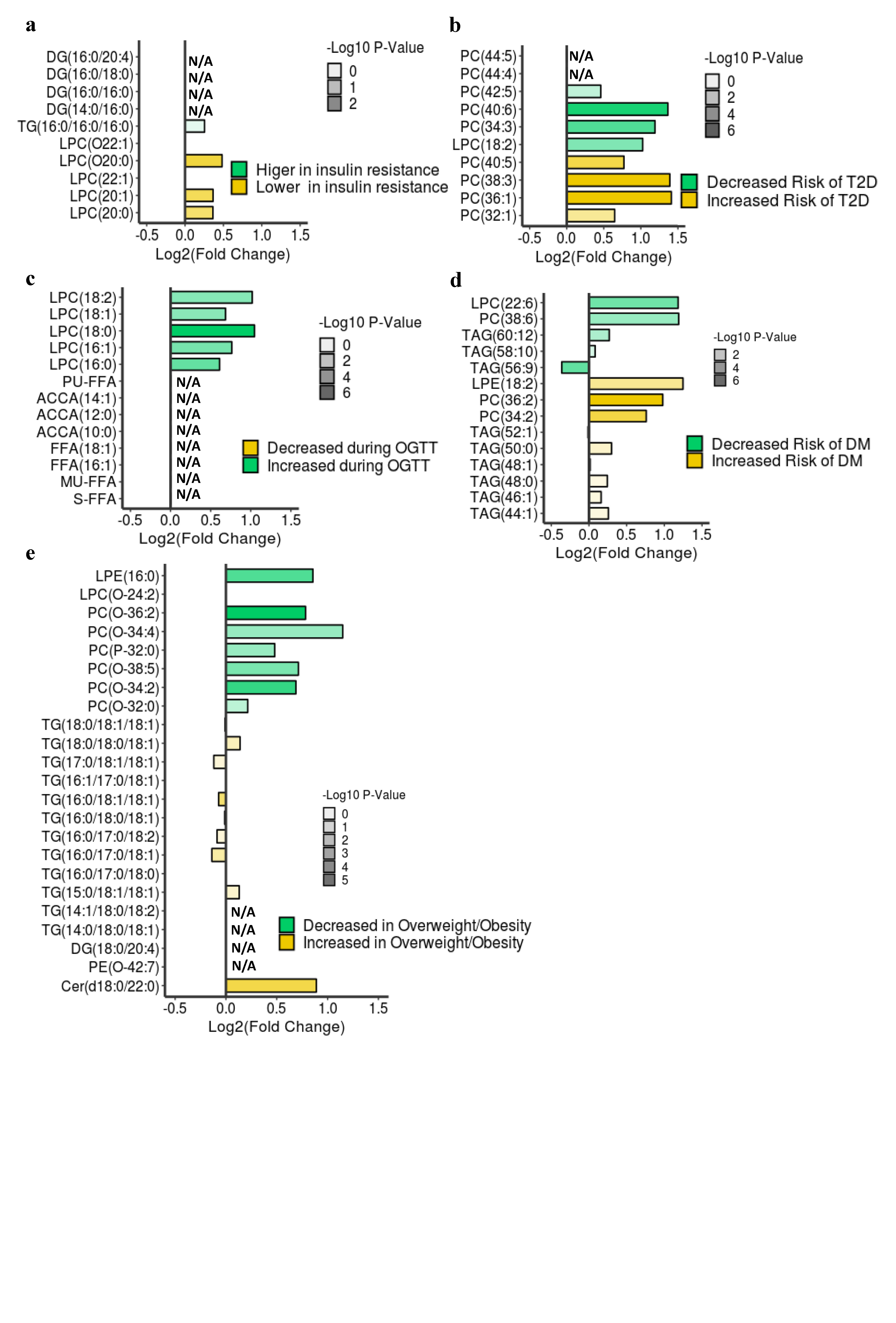

Supplement: Supplementary file 14 — Figure S7. Comparing of the lipidic programs in iWAT from cold-treated models and the plasma from insulin-resistant and obese/overweight patients. (a) The intensity of the lipid species that were reported to be significantly changed in insulin resistance patients. Green columns represent higher lipid species in insulin resistance patient. Yellow columns represent higher lipid species in insulin resistance patient. (b) The intensity of the lipid species that were reported to be markers for T2D. Green columns represent lipid species that might decrease risk of T2D. Green columns represent lipid species that might increase risk of T2D. (c) The intensity of the lipid species that were reported to be significantly changed during OGTT. Green columns represent lipid species that were increased during OGTT. Yellow columns represent lipid species that were decreased during OGTT. (d) The intensity of the lipid species that were reported to be markers for DM. Green columns represent lipid species that might decrease risk of DM. Green columns represent lipid species that might increase risk of DM. (e) The intensity of the lipid species that were reported to be significantly changed in overweight/obesity. Green columns represent decreased lipid species in overweight/obesity. Yellow columns represent increased lipid species in overweight/obesity. The transparency of each bar is proportional to the significance values, which are displayed as -log10 (P-value). (JPG 1667 kb) [file 12915_2019_693_MOESM14_ESM.jpg]
